# Coronavirus, the King Who Wanted More Than a Crown: From Common to the Highly Pathogenic SARS-CoV-2, Is the Key in the Accessory Genes?

**DOI:** 10.3389/fmicb.2021.682603

**Published:** 2021-07-14

**Authors:** Nathalie Chazal

**Affiliations:** Institut de Recherche en Infectiologie de Montpellier (IRIM), Université de Montpellier, CNRS, Montpellier, France

**Keywords:** SARS-CoV-2, origin, biology, accessory proteins, evolution

## Abstract

Severe acute respiratory syndrome coronavirus 2 (SARS-CoV-2), that emerged in late 2019, is the etiologic agent of the current “coronavirus disease 2019” (COVID-19) pandemic, which has serious health implications and a significant global economic impact. Of the seven human coronaviruses, all of which have a zoonotic origin, the pandemic SARS-CoV-2, is the third emerging coronavirus, in the 21st century, highly pathogenic to the human population. Previous human coronavirus outbreaks (SARS-CoV-1 and MERS-CoV) have already provided several valuable information on some of the common molecular and cellular mechanisms of coronavirus infections as well as their origin. However, to meet the new challenge caused by the SARS-CoV-2, a detailed understanding of the biological specificities, as well as knowledge of the origin are crucial to provide information on viral pathogenicity, transmission and epidemiology, and to enable strategies for therapeutic interventions and drug discovery. Therefore, in this review, we summarize the current advances in SARS-CoV-2 knowledges, in light of pre-existing information of other recently emerging coronaviruses. We depict the specificity of the immune response of wild bats and discuss current knowledge of the genetic diversity of bat-hosted coronaviruses that promotes viral genome expansion (accessory gene acquisition). In addition, we describe the basic virology of coronaviruses with a special focus SARS-CoV-2. Finally, we highlight, in detail, the current knowledge of genes and accessory proteins which we postulate to be the major keys to promote virus adaptation to specific hosts (bat and human), to contribute to the suppression of immune responses, as well as to pathogenicity.

## Coronaviruses: Taxonomy and Historically Perspectives

**FIGURE 1:**
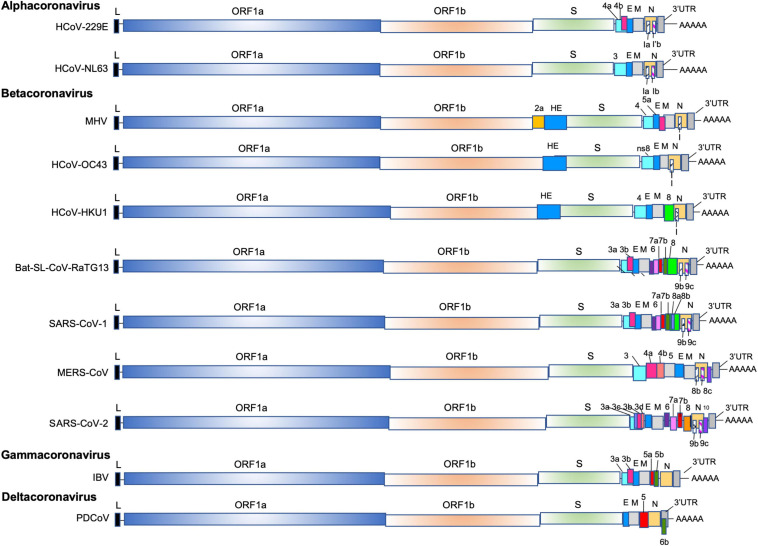
The genomes of alpha-CoVs (HCoV-229E, HCoV-NL63), beta-Covs (MHV, HCoV-OC43, HCoV-HKU1, Bat-SL-RaTG13, SARS-CoV-1, MERS-CoV and SARS-CoV-2), delta-Covs (IBV), and gamma-CoVs (PDCoV). Coronaviruses contain a positive-sense, single-stranded RNA [ssRNA (+)] genome of 27–32 kb. The 5′-terminal two thirds of the genome encode a polyprotein pp1a and pp1b which are further cleaved into 16 nps that are involved in genome transcription and replication. The 3′ terminus encodes structural proteins: envelope glycoprotein (S), envelope (E), membrane (M), and nucleocapsid (N). In addition, accessory genes species-specific are interspersed or embedded as an alternative ORF within another genes (the ORF I gene of MHV, the Ia and Ib gene). The SARS-CoV-1 (SARS-CoV-1 reference sequence AY274119) possess 9 ORFs (ORF3a, 3b, 6, 7a, 7b, 8a, 8b, 9b, and 9c), the MERS (MERS-CoV reference sequence NC_019843) retains 6 ORFS (ORF3, 4a, 4b, 5, 8b, and 8c), and the SARS-CoV-2 (SARS-CoV-2 reference sequence NC_045512) has 11 ORFS (ORF3a, 3b, 3c, 3d, 6, 7a, 7b, 8, 9b, 9c, and 10).

Inside the *Nidovirales* order and in the sub-order *Cornidovirineae*, the highly diverse *coronaviridae* family of enveloped positive-sense single-stranded RNA viruses is divided in two sub-families *Letovirinae* and *Orthocoronavirinae.* The sub-family *Orthocoronavirinae* is composed of four genera: *alphacoronaviruses* (fourteen sub-genera), *betacoronavirus* (five sub-genera), *gammacoronavirus* (three sub-genera) and *deltacoronavirus* (three sub-genera) (Figure 1). The human coronaviruses HCoV-229E and HCoV-NL63 are members of *alphacoronavirus* (alpha-CoVs); the Murine coronavirus (previously named Mouse Hepatitis Virus, MHV), the two human coronaviruses HCoV-OC43 and HKU1, the Severe Acute Respiratory Syndrome (SARS)-Cororonavirus (CoV)-1, the Middle East Respiratory Syndrome Coronavirus (MERS)-CoV, the bat SARS-CoV-like coronaviruses and the SARS-CoV-2 are included in the *betacoronavirus genus* (beta-CoVs)*;* the Goose coronavirus CB17, the Avian coronavirus, the Avian coronavirus 9203, the Duck coronavirus 2714 and the Beluga whale coronavirus 1 are members of *gammacoronavirus* (gamma-CoVs); and the porcine deltacoronavirus (PVCoV) is a part of the *deltacoronaviruses* (delta-CoVs). Whereas alpha-CoVs and beta-CoVs exclusively infect mammalian species, gamma-CoVs and delta-CoVs have a wider host range.

The discovery of the first coronavirus occurred in the first third of the 20th century. In 1931, in the North Dakota, a new type of upper respiratory tract disease was described among chickens (Schalk and Hawn, 1931). Five years later, the virus was isolated and named Infectious Bronchitis Virus (IBV, later renamed as Avian coronavirus) (Beach and Schalm, 1936). Successively, in 1946 and independently in 1949 and 1951, two other animal coronaviruses were described: the transmissible gastroenteritis virus in pigs (later named transmissible gastroenteritis coronavirus, TGEV) (Doyle and Hutchings, 1946) and the murine hepatitis virus (MHV) (Cheever et al., 1949; Gledhill and Andrewes, 1951). In 1965 and 1966, for the first time, two human coronaviruses were isolated from the nasal washings of a male child collected 5 years earlier and from the respiratory tract of medical students with cold collected in 1962, respectively, and named: B814 (B814 was no longer study) and 229E (later renamed human coronavirus-229E, HCoV-229E) (Tyrrell and Bynoe, 1965; Hamre and Procknow, 1966). Based on transmission electron microscopy studies and making comparison between (IBV, MHV and HCoV-229E), by mid-1967, in London, June Dalziel Almeida and David Tyrrell recognized that these three viruses were structurally similar and decided collectively to give the name of coronavirus as all those viruses were characterized by corona-like projections (spikes) on their surface (Almeida and Tyrrell, 1967). In 1967, Kenneth McIntosh and co-workers characterized new human coronaviruses called the human “IBV-like virus” and later renamed human coronavirus-Organ Culture (OC)OC38 and -OC43 (HCoV-OC43) (McIntosh et al., 1967a,b). More recently, the diversity of *Coronaviridae* has greatly improved and a numerous of novel coronaviruses have been discovered in human: the SARS-CoV-1 in 2003, the HCoV-NL63 in 2004 (van der Hoek et al., 2004), the HCoV HKU1 in 2005 (Woo et al., 2005), the MERS-CoV in 2012 (Zaki et al., 2012), but also in a vast variety of animals especially several bat species throughout Africa, America, Asia and Europe (Poudel et al., 2020) until the discovery of SARS-CoV-2 in late 2019, which adds to the list of six known HCoVs associated with different disease phenotypes, from common to highly pathogenic.

## From Common to Highly Pathogenic Human Coronaviruses: Outbreaks and Physiopathology of Coronaviruses Infection

As we saw above, coronaviruses are broadly distributed among humans, and animals (bats, birds, cats, dogs, pigs, mice, horses, whales etc.) causing respiratory, enteric, hepatic, or neurological disorders with variable severity. Animal coronaviruses were mainly studied during several decades because they cause economically significant respiratory and gastrointestinal diseases in domestic animals (20,000 pigs killed by an HKU2-related coronavirus) (Guy, 1998; Saif, 2004; Zhou et al., 2018) and because they provide unique models to study coronavirus pathogenesis. On the other hand, during a long period, the human coronavirus research remained more confidential. However, soon after the first isolation of HCoV-229E and HCoV-OC43, their cellular receptors were characterized. The HCoV-229E was shown to bind to the cellular receptor aminopeptidase N (APN), while the HCoV-OC43 was reported to utilize HLA class I molecule or sialic acids for entry (McIntosh et al., 1967a; Collins, 1993; Krempl et al., 1995). Later, the HCoV-NL63 was found to use angiotensin-converting enzyme–related carboxypeptidase (ACE2) (van der Hoek et al., 2004) and the HKU1, to exploit the HLA class I molecule or sialic acids as receptors (Woo et al., 2005; Esper et al., 2006; Lau et al., 2006; Sloots et al., 2006; Vabret et al., 2006; Chan et al., 2009; Hulswit et al., 2019). As these four HCoVs infect the respiratory tract and are known to be mostly responsible for common colds, the knowledges of these human viruses have only progressively evolved.

However, the field of human coronaviruses research took an abrupt turn when, in late 2002, a new epidemic human disease emerged in southeast China (Guangdong Province), Hong-Kong, Vietnam and subsequently spread to 32 countries, which was caused by an unknown coronavirus. This new disease was named SARS and the causative agent was quickly identified and named SARS-CoV-1. SARS-CoV-1 was transmitted by direct contacts with patients, but also *via* aerosol droplets and contaminated stool (Weinstein, 2004). Air travel by infected individuals quickly spread the disease resulting in the first severe epidemic of the twenty-first century. Over a 6-month period this epidemic led to 8098 illnesses and 774 deaths worldwide (mortality = 9.7%) (Petersen et al., 2020).

Lately in 2012, a new coronavirus from a man suffering of a severe respiratory syndrome was isolated in Saudi Arabia. This new coronavirus, named MERS-CoV that uses the dipeptidyl peptidase 4 (DPP4 also named CD26) as receptor, was responsible of an epidemic disease mostly in Arabic peninsula (Saudi Arabia) leading to 2,494 illnesses and 858 deaths (mortality = 34%) (Alfaraj et al., 2019).

In late 2019, a new strain of coronavirus named SARS-CoV-2, emerged in Wuhan, the capital of China’s Hubei province, and the associated illness was named Coronavirus Disease 2019 (COVID-19) (Wu et al., 2020; Zhou et al., 2020c). Following the first case in Wuhan, the infection spread rapidly until March 11, 2020 when the World Health Organization (WHO) declared COVID-19 as a pandemic. SARS-CoV-2 is transmitted mainly through droplets, close contacts and aerosols. The COVID-19 has considerable global economic and severe health impacts around the world (Ahmad et al., 2020; Munir et al., 2020). As observed in patients infected with SARS-CoV-1 and MERS-CoV, the COVID-19 symptoms are fever, dry cough, fatigue, lymphopenia and can also lead to gastrointestinal symptoms or/and acute respiratory distress syndrome. In all three cases a strong and uncontrolled cytokine release syndrome (“cytokine storm”), responsible of the acute respiratory distress, can be observed in patients (Moore and June, 2020; Tay et al., 2020). As SARS-CoV-1 (Hamming et al., 2004; Jia et al., 2005; Xu et al., 2020), the SARS-CoV-2 uses ACE2 as entry receptor and employs the cellular serine protease TMPRSS2 for envelope protein Spike (S) priming (Li et al., 2003; Kuba et al., 2005; Matsuyama et al., 2010; Glowacka et al., 2011; Shulla et al., 2011; Hoffmann et al., 2020). As the ACE2 receptor is widely expressed in the arterial and endothelial cells, the arterial smooth muscle cells and especially highly expressed in the lung, renal, cardiovascular, gastrointestinal tissues, but also in hematopoietic cells as macrophages and monocytes, SARS-CoV-1 was shown to infect primary human monocytes, macrophages and but also DCs (Cheung et al., 2005; Gu et al., 2005; Law et al., 2005a; Tseng et al., 2005; Yilla et al., 2005). However, SARS-CoV-1 replication was also described to be abortive in macrophages and the virus was shown be phagocytosed by these cells (Yilla et al., 2005). MERS-CoV was found to infect monocyte-derived macrophages and DCs, although low replication was observed in these cells (Zhou et al., 2015; Tynell et al., 2016). For SAR-CoV-2, as the DCs, residing notably in the lungs, express at the surface the ACE2, these cells can be directly infected by SARS-CoV-2. Moreover, as for the SARS-CoV-1, the SARS-CoV-2 can also invade host cells via the atypic route of CD147-spike protein (SP) (Wang et al., 2020). Surprisingly, the monocytes obtained from the COVID-19 positive patients are found to be actively expressing ACE2 receptor, suggesting the possibility of direct infection of the monocytes thereby leading to abrogated viral replication and a delayed type I IFN signaling (Acharya et al., 2020). On the other hand, morphological and functional differences in monocytes derived from COVID-19 patients in comparison with monocytes from healthy donors were also observed (Zhang et al., 2021a). Recently, the SARS-CoV-2 infection of ACE2-positive macrophages, present in the spleen and lymph nodes together with alveolar type II pneumocytes and macrophages, were reported (Feng et al., 2020; Xu et al., 2020; Zhang et al., 2020b). SARS-CoV-1, MERS CoV and SARS-CoV-2 are cytopathic viruses, which induce notably lung cell death and trigger immune response (Vabret et al., 2020). Macrophages and monocytes, recruited in response to the infection, release cytokines [interferons I/III (IFN-I/III)], but also proinflammatory Tumor Necrosis Factor alpha (TNF-α), Interleukin-1 (IL-1), IL-6 and IL-18 to prime adaptative T and B cell immune responses (Tay et al., 2020). In most cases, this process is capable of resolving the infection, the immune response declines and patients recover. However, some patients develop a severe disease (3–10% of subjects requires hospitalization, with up to 20% of them experiencing severe disease and a high mortality rate), as it has been previously described for the SARS-CoV-1 and the MERS-CoV infections (Channappanavar et al., 2016, 2019), which is often associated with an impaired type I IFN signature that is blocked by multiple processes at various stages of the IFN signaling pathway (Sa Ribero et al., 2020; Vabret et al., 2020; Xia et al., 2020). This strong antagonism is supposed to assist viral replication, but also to induce an increased release of pyroptosis products that can further bring an aberrant inflammatory response. Together, in addition to the direct damage resulting from the virus replication, the dysregulated pro-inflammatory response can mediate damages in the lung, but also can cause septic shock and induce a multi-organ failure. Patients with severe COVID-19, exhibited, as we mentioned above, an immune dysfunction (dysregulation of T-cells, B-Cells, and innate immune-cells). They present a significantly impaired and delayed secretion of IFN-I and IFN-III compared with flu patient and higher blood plasma levels of IL-2, IL-7, IL-10, granulocyte colony-stimulating factor (G-CSF), IFN gamma-induced protein 10 (IP-10), monocyte chemoattractant protein 1 (MCP1), macrophage inflammatory protein 1α (MIP1α), TNF-α, IL-6, and also show, a higher percentage of CD14 + CD16+ inflammatory monocytes in peripheral blood that secrete inflammatory cytokines contributing to the cytokine storm (Blanco-Melo et al., 2020; Huang et al., 2020; Vabret et al., 2020; Zhou et al., 2020a,d). Together, as with SARS-CoV-1, the clinical manifestation of SARS-CoV-2 results from a dysregulated immune response in patients. SARS-CoV-2 can antagonize type I interferon (IFN-I) production, attenuate activation of the IFN-I signaling pathway, disrupt antigen presentation, increase the production of proinflammatory mediators, in some cases resulting in worsening of the disease. Therefore, understanding the molecular interactions between SARS-CoV-2 and the host defense machinery is crucial for improving treatment interventions. Among several actors, accessory proteins, have been found to modulate the host immune response and to be involve in the inflammatory response. To explore the role played by SARS-CoV-2 accessory proteins in the physiopathology of SARS-CoV-2, additional study remains necessary and especially on the origin highly pathogenic coronaviruses.

## Origin of Human Coronaviruses: SARS-CoV-1, MERS-CoV, and SARS-CoV-2

**FIGURE 2:**
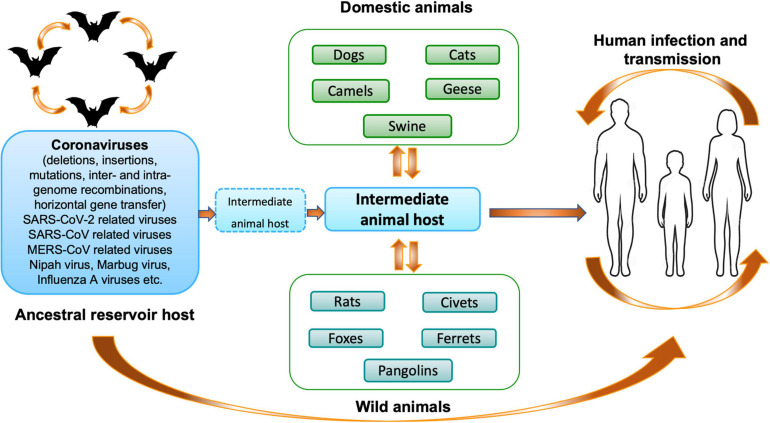
Origin and transmission of coronaviruses. Schematic representation of the origin and transmission of coronaviruses, as well as SARS-CoV-2, between hosts and humans (Mahtarin et al., 2020).

Different phylogenetic studies have shown that rodents and bats may act as the main reservoir of most alpha-CoVs and beta-CoVs, while birds may be the source of gamma- and delta-CoVs (Su et al., 2016). To date, seven human-CoVs that have zoonotic origins or circulate in animals are known: two alpha-CoVs (HCoV-229E and HCoV-NL63) and five beta-CoVs (HCoV-OC43, HCoV-HKU1, SARS-CoV-1, MERS-CoV, and SARS-CoV-2). They share the common characteristic of having a bat coronavirus as a direct or indirect ancestor (Banerjee et al., 2019b; Cui et al., 2019; Letko et al., 2020). Since the emergence of SARS-CoV-1, MERS-CoV and SARS-COV-2, it becomes clear that bats are probably one of the main reservoirs of CoVs (Figure 2). In 2017, a study, conducted by the Goldstein’s team, on 12,333 bats representing 282 species from 20 countries in Asia, Africa and Latin America described that 9% of bats carried at least one of 91 distinct coronaviruses. They estimated that there are at minimum 3,204 coronaviruses in bats, supporting the hypothesis that bats can be infected by multiple coronaviruses and that recombination events may occurred between several coronaviruses present in these animals (Anthony et al., 2017b). Interestingly, the authors observed that host switching events (inter-genus or family switching) were proportionally lower in Latin America compared with Africa and Asia without understanding why, suggesting that Africa and Asia are highly susceptible geographic zones for zoonotic emergence.

The emergence of HCoV-OC43 in humans was proposed to occur at the end of the 19th century, when a pandemic of respiratory disease was recorded in humans (Vijgen et al., 2005, 2006). This emergence was suggested to be linked to a host-switching event from an animal-CoV to a human. Indeed, HCoV-OC43 apparently emerged from a bovine coronavirus (BCoV) spillover from a single cross-species transmission event that gave rise to a human-only virus (Vijgen et al., 2005, 2006; Lau et al., 2011). However, the recombination theory from different animal coronaviruses was also retained to promote the emergence of this variant. Indeed, recently a novel canine respiratory coronavirus strain was characterized, which likely resulted from recombination between a Chinese canine respiratory coronavirus and BCoV (Lu et al., 2017).

In 2010, a bat coronavirus termed Appalachian Ridge CoV (ARCoV-2) was detected in North American tricolored bat (*Perimyotis subflavus*) in the United States and showed close relationship with HCoV-NL63 with an estimated divergence of ∼550 years ago (Donaldson et al., 2010; Huynh et al., 2012; Banerjee et al., 2019b). In 2007, sequences related to HCoV-229E were obtained from captive alpacas (*Vicugna pacos*) in California (Crossley et al., 2010). The single virus strain, termed Alpaca coronavirus (A-CoV), was sequenced and found to be highly related to known HCoV-229E strains. In 2009, close relatives of HCoV-229E were detected in bats from Ghana (Pfefferle et al., 2009b). Later on, a phylogenetic analysis suggested that the HCoV-229E possess an ancestral origin in *hipposideridae* bats from Ghana and that camelids could be potential intermediate hosts (Corman et al., 2015). Phylogenetic analyses of complete genomes placed the dromedary-camel-associated viruses in close relationship to HCoV-229E, while the A-CoV clustered with dromedary-camel viruses. This close proximity was explained by the fact that dromedaries are, on occasion, kept along with alpacas in husbandries and zoological gardens. Interestingly, seropositive samples to HCoV-229E of camels were found in Arabian Peninsula and Africa supporting the notion that HCoV-229E has an ancestral origin in bats while camelids serve as a zoonotic natural reservoir for human transmission (Corman et al., 2016). More recently, different bat-CoVs, closely related and ancestor of HCoV-NL63, were identified in *Triaenops* bats from Kenya. Interestingly, these NL63-like viruses have acquired, by a recombination event, a 229E-like S gene from 229E-like viruses circulating in *Hipposideros* bats (Tao et al., 2017). With HKU1, to date, no related virus has been isolated from any animal species. However, as HCoV-HKU1 was depicted to be most closely related to MHV and a rat coronavirus (rat sialodacryoadenitis virus), in consequence it cannot be excluded that the hosts of the ancestral viruses are carried by rodents (Woo et al., 2005; Lau et al., 2015b).

The origin of SARS-CoV-1 was first shown by the isolation and the identification of closely related viruses in Himalayan palm civets and also raccoon dog at a live-animal market in Guangdong, China (Guan, 2003). A very high genome sequence identity (99%) was described between the SARS-CoV-1 isolated from civets and SARS-CoV-1 from humans, supporting the notion that SARS-CoV-1 is of animal origin. However, subsequent studies showed that palm civets on farms and in the field were largely free from SARS-CoV-1 infection. These latest results have thus made possible to propose that palm civets, rather than a natural reservoir, play the role of an intermediate host. In subsequent studies, among different bat populations, two teams reported the discovery of CoVs related to SARS-CoV-1 in horseshoe bats (genus *Rhinolophus*) suggesting that SARS-CoV-1 could have spilled over into humans directly from bats while the civet and the raccoon dog, were incidental hosts (Lau et al., 2005; Li et al., 2005). This hypothesis was reinforced by the isolation, during surveillance campaign, of bats coronaviruses closely matching the human SARS-CoV-1 (Ge et al., 2013; Kuehn, 2013). These viruses were called “SARS-CoV-like viruses” (SL-CoV) or “SARS-CoV-related viruses”. Chinese horseshoe bats (*Rhinopholus*), belonging to the family *Rhinolophidae* of the order *Chiroptera* under Microchiroptera (microbats), is an insectivorous species widely distributed notably in Chinese forests, however, SL-CoVs were also discovered in *Rhinolophus* from other geographical origins (European, African, and Southeast Asian countries). Indeed, SL-CoVs were also discovered in *rhinolophids* from Slovenia, Bulgaria, and Italy in Europe (Drexler et al., 2010; Rihtaric et al., 2010; Balboni et al., 2011). In Africa, novel beta-CoVs related to SARS-CoV-1 were also detected in bats (*Hipposideros* and *Chaerephon* genus) from Ghana, Kenya, and Nigeria (Pfefferle et al., 2009b; Tong et al., 2009; Quan et al., 2010; Hu et al., 2015). However, compared with Asian and European bat SL-CoVs, these viruses were phylogenetically distant to SARS-CoV-1. Overall, it was postulated that the direct ancestor of SARS-CoV-1 may have arisen from sequential recombination events between the precursors of the bat SL-CoVs prior to spillover directly to humans or by an intermediate host and that humans were exposed to bat SL-CoVs prior to the SARS-CoV-1 epidemic 2002–2003. Indeed, it has been suggested that the ancestor of civet SARS-CoV-1 probably acquired the Open Reading Frame 8 (ORF8) from *Rhinopholus ferrumequinum* SL-CoVs by recombination (Lau et al., 2015a) and seroprevalence studies described a stronger antibodies response against SL-CoV isolated from Himalayan palm civet rather than human SARS-CoV-1 in sera of healthy individuals in Hong-Kong in 2001 (Zheng et al., 2004).

Concerning MERS-CoV, neutralizing antibodies against this virus were found in the sera of dromedary-camels in the Middle East (Reusken et al., 2013) and in several African countries (Chan et al., 2015). A MERS-CoV strain, identical to the human MERS-CoV, was also isolated from the nasal swabs of dromedary-camels (Raj et al., 2014), suggesting that dromedary-camels are the natural reservoir of MERS-CoV. However, various coronaviruses that are phylogenetically related to MERS-CoV, but phylogenetically distant from humans and dromedary-camels, have also been found in different species of bats, including Egyptian tomb bats (Al-Tawfiq and Memish, 2020), African bats (Ithete et al., 2013; Corman et al., 2014a; Anthony et al., 2017a), Italian bats (Lelli et al., 2013; Moreno et al., 2017), and Chinese bats (Yang et al., 2014), suggesting that these animals are the ancestral reservoir. Thus, it is possible that other, yet unidentified strains of bat viruses, are circulating in nature and may have directly contributed to the emergence of MERS-CoV in camels less than 30 years ago and later in humans (Reusken et al., 2013; Corman et al., 2014b; Müller et al., 2014; Alshukairi et al., 2018; Al-Tawfiq and Memish, 2019; Kiyong’a et al., 2020). Again, many recombination events, involving the exchange of genetic elements between different viral ancestors, including those isolated from dromedary-camels and bats, are suspected.

Two main scenarios that can plausibly explain the origin of SARS-CoV-2 are actually proposed (Figure 2): (i) natural selection in an animal host before zoonotic transfer; and (ii) natural selection in humans following zoonotic transfer (Andersen et al., 2020; Lam et al., 2020a; Xiao et al., 2020; Zhou et al., 2020c). SARS-CoV-2, closely related to a bat-SL-CoV-RaTG13 (96.2% nucleotide identity), which had previously been found in 2013 in bats *Rhinolophus affinis* in Pu’er (Yunnan Province, China), a bat-SL-CoV-RmYN02 collected in *Rhinolophus malayanus* in the Yunnan Province in 2019 (93.3% nucleotide identity), a bat-SL-CoV-ZXC21/ZC45 related (87.6–87.8% nucleotide identity) found in bats *Rhinolophus pusillus* from Zhoushan, China, has emerged (Hu et al., 2018; Zhang and Holmes, 2020; Zhou et al., 2020b,c). Soon after the first detected case of SARS-CoV-2 infection in humans, it was hypothesized that the first case of animal-to-human transmission of SARS-CoV-2 was linked to an animal intermediate host, which would have been among the wildlife species sold on the Huanan market in Wuhan (Huang et al., 2020). Several sequencing studies have suggested that the pangolins could harbor ancestral beta-CoVs related to SARS-CoV-2 (85–92% nucleotide sequence homology with SARS-CoV-2) (Liu et al., 2019; Lam et al., 2020b). Indeed, Pangolin-SARS-CoV-2-related-Cov was found to share a very similar Receptor Binding Domain (RBD) with SARS-CoV-2, (97.4% amino acid sequence identity), while the RBDs of bat-SL-CoV-RaTG13 were more divergent albeit higher degree of sequence homology (Lam et al., 2020b). However, due to the sequence divergence between SARS-CoV-2 and pangolin SARS-CoV-2-related beta-CoVs, the origin of the virus has not been determined conclusively and the evolutionary pathway of SARS-CoV-2 in bats, pangolins or other mammals remains to be established. Another proposal is in favor of multiple recombination events between a pangolin SARS-CoV-2-related beta-CoV (Xiao et al., 2020) and the bat-SL-CoVs in a third wild animal species. Since the beginning of the SARS-CoV-2 pandemic, extensive discussions regarding its origin took place. The molecular specificity of the SARS-CoV-2, however, indicates that the virus is not the product of purposeful manipulation. Indeed, the RBD sequence of SARS-CoV-2 is not optimal such as the SARS-CoV-1, suggesting that a natural selection is more probable. Another hypothesis is that SARS-CoV-2 could have originated from a bat virus that has been adapted to experimental animal models or laboratory cells and that has escaped from a laboratory. In any case, further research is required before a final decision can be made on the exact origin of the SARS-CoV-2. Bats are presumed reservoirs of diverse coronaviruses including progenitors of respiratory syndrome: SARS-CoV-1, MERS-CoV and SARS-CoV-2, but also other coronaviruses as well as viruses from different families. The complete characterization of the bat immune system is a major challenge in understanding the adaptation of coronaviruses.

## Bat and Coronaviruses: Immune Response

Bats, which harbor a large number of zoonotic viruses per species, are animals that host the larger number of zoonotic viruses after rodents, and they are the second most diverse mammalian order on earth that are distributed across every continent except Antarctica (Luis et al., 2013). The order *Chiroptera* is complex and consists of 22 families over genera and 1,400 species of bats that are distributed across most continent (Fenton and Simmons, 2015). This order is divided in two suborders: *Yinpterochiroptera* and *Yangochiroptera*. Within these suborders, bat species display considerable diversity in diets, sizes, morphologies and ecological niches. Until now, 28 different virus families (DNA and RNA viruses) have been identified in bats. Among these viruses, several RNA viruses are zoonotic viruses (coronaviruses, henipaviruses, lyssaviruses, and filoviruses). Although they do not induce serious clinical symptoms and pathogenesis in bats, as far as we know, they represent a danger of great importance for humans and veterinary health (Ge et al., 2013; Ithete et al., 2013; Anthony et al., 2017a; Hu et al., 2017). The basis of bat resistance to viral infections is still very poorly understood. Some argue that this resistance is related to their high-energy metabolism required during their flight that would induce a high body temperature and mimic the fever phenomenon observed during the activation of the immune response, allowing a reduction of virus replication (O’Shea et al., 2014; Brook and Dobson, 2015). However, the replication of filoviruses (Marburg virus and Ebola virus), in cell lines derived from several bat species, was found to be also effective at 37°C or 41°C, contradicting this hypothesis (Miller et al., 2016). The bat resistance is likely more related, for a large part, to the presence of a specific, but not universal, immune system. In recent years, significant efforts in the study of the biology of some bat species has been made and several characterizations of bat innate immunity have given some insights into the differences between bat and human immune responses. To date, the genomes and transcriptomes of at least 18 bat species have been completed (Hawkins et al., 2019; Jebb et al., 2020). These analysis revealed that only a small proportion of the innate immune system homolog genes (in human immune-related genes represent 7% of the genome while in bat they represent only 2.75–3.5% of the transcribed genes) is detected in the bat genome (Egyptian fruit bat: *Rousettus aegyptiacus* and black flying fox: *Pteropus alecto*) (Papenfuss et al., 2012; Lee et al., 2015). Moreover, the analysis of the *Pteropus alecto* transcriptome has also allowed the identification of a significant proportion of bat-specific transcripts without homology with known annotated transcripts, which may be involved in the immune response (Papenfuss et al., 2012). Even if innate immune response has been poorly depicted in bats, the bat cells from multiple species were shown to upregulate efficiently type I IFNs and IFN-Stimulated Genes (ISGs) in response to poly(I:C) treatment, suggesting that a sensing machinery is conserved in bat cells (Omatsu et al., 2008; Crameri et al., 2009; Cowled et al., 2012; Banerjee et al., 2016, 2017, 2020). Thus, full-length transcripts for Pattern Recognition Receptors (PRRs) as Toll-Like Receptors-1 (TLR-1) to TLR-10 have been detected and a TLR-13 pseudogene have been identified in *P. alecto* (Cowled et al., 2011). However, differences have be found in TLRs sequences between bats and humans, but also within the bat species (Schad and Voigt, 2016). Contrary to TLRs, cytosolic RNA sensors (Retinoic-acid Inducible Gene I: RIG-I and Melanoma Differentiation-Associated gene *5*: MDA-5) have been shown to be conserved in most bat genomes or transcriptomes (Cowled et al., 2012; Papenfuss et al., 2012; Banerjee et al., 2017). In human cells, virus recognition, and related-signaling pathways, leads to phosphorylation of transcription factors like Interferon Regulatory Factor 3 and 7 (IRF3 and IRF7). Bat IRF3 sequences has been found evolutionarily distinct from their mammalian counterparts (Banerjee et al., 2019a), but functional studies indicate that IRF3 from *Eptesicus fuscus* cells mediates antiviral signaling in response to MERS-CoV infection or poly(I:C) stimulation, while IRF7 is induced following poly(I:C) stimulation in both *E. fuscus* and *P. alecto* cells (Zhou et al., 2014; Banerjee et al., 2017). Interestingly, IRF7 mRNA has been found to be constitutively expressed in *P. alecto* cells and to have a ubiquitarian tissue distribution compared to its human counterpart (Zhou et al., 2014; Banerjee et al., 2019a). RIG-I-like receptors (RLRs) are intracellular sensors for viral RNAs that activate Mitochondrial AntiViral Signaling protein (MAVS) leading to nuclear translocation of NF-kB and IRF3 for the induction of type I IFNs (Seth et al., 2005). Functional MAVS signaling has been established to be present in the straw-colored fruit bat (*Eidolon helvum*) and in the horseshoe bat (*Rhinolophus sinicus*). However, downstream signaling pathways and molecules involved in MAVS-mediated signaling have not yet been characterized in bats. IFN response is critical to restrain virus propagation. Constitutive expression of IFN-α transcripts and associated IFN-stimulated genes (ISGs) have been detected in unstimulated *P. alecto* cells (a contraction of the IFN locus with only 3 functional IFN-α genes exists in this bat genome compared with 7–18 IFN-α loci in other mammals and in spite of everything a constitutive and ubiquitous expression is observed in these specific bats), but not in primary cells from *R. aegyptiacus*, which have a higher expression of IFN-α/β receptors (IFNAR1 and IFNAR2) and several ISGs (Zhou et al., 2016; Pavlovich et al., 2018). In *Pteropus vampyrus* genome, coding sequences for three type III IFNs have been described, but only two of them were found to be transcribed in the closely related bat *P. alecto* (Zhou et al., 2011). Altogether, these data suggest that species-specific differences in IFN responses exist in bats. In addition to these characteristics related to the species of bat, another parameter must be considered. Indeed, viral infections of bat cells with viruses from several families have been shown to either induce or inhibit IFN production. Indeed, MERS-CoV that inhibits antiviral IFN expression in human cells, induces an increase in IFN-β and 2–5 -OligoAdenylate Synthase 1 (OAS1) transcripts in MERS-CoV-infected *E. fuscus* cells (Banerjee et al., 2019a) and a transient upregulation of Myxovirus resistance protein 1 (Mx1), ISG56 and Regulated on Activation Normal T cell Expressed and Secreted (RANTES) in infected Jamaican fruit bats (*Artibeus jamaicensis*) cells (Munster et al., 2016), whereas Nipah virus infection has been shown to inhibit IFN production in infected-*P. alecto* cells (Virtue et al., 2011). IFN signaling in *P. alecto* cells is dependent on IFNAR2 (Zhang et al., 2017). It has not yet been established how many ISGs are induced in different bat cells, but considering the vast bat species diversity, it is likely that bat species express different ISGs. Poly(I:C) stimulation was shown to induce the expression of transcripts for MDA5, RIG-I, radical *S* adenosylmethionine domain-containing 2 (RSAD2), IRF7, OAS1, IFN-inducible protein 6 (IFI6) and Mx1 in *E. fuscus* cells (Banerjee et al., 2017). Expression of Mx1, OAS1 and Protein Kinase R (PKR) transcripts was also found to be induced in *P. alecto* cells (Zhou et al., 2013). Even if the expression of Mx1 from six different bat species in human embryonic kidney (HEK 293T) cells was found to efficiently reduce Ebola virus and influenza A virus replication (Fuchs et al., 2017), the expression of atypical ISGs was also described in *P. vampyrus* (RND1, SERTA-domain containing 1 (SERTAD1), ChaC glutathione specific gamma-glutamyl cyclotranferase 1 (CHAC1), MORC3) (Glennon et al., 2015; Banerjee et al., 2020). While keeping IFN response or not, bats seem to support unique adaptations to control inflammation, as far as we know, thus limiting tissue damages and pathologies. This apparent lack of a strong inflammatory response in virally-infected bat immune cells has been linked, so far, to two features: (i) a dampened activation of the NLRP3 inflammasome in bat immune cells compared to human immune cells without impacting the overall viral loads (Ahn et al., 2019) and (ii) an interaction of a transcription repressor of the NF-kB family, c-Rel, with the putative c-Rel motif located in the TNF- promoter in the big brown bat (*E. fuscus*) (Banerjee et al., 2017).

Even if studies on adaptative immunity of bats have been so far limited, some information are available. Indeed, the antibody response in bats is poorly known, however, the detection of transcripts of the four major subclasses of antibodies such as IgA, IgE, IgG, and IgM has been performed in bats cells (Baker et al., 2010; Wynne et al., 2013). Experiments on *R. aegyptiacus* with Marburg virus showed variability in antibody response. One study reported a detectable antibody response for up to 3 months (Schuh et al., 2017), while another study reported a detectable antibody response for up to 11 months (Storm et al., 2018). Moreover, studies of sera of convalescent *R. aegyptiacus* bats challenged with Ebola virus, Marburg virus and Sosuga virus, demonstrated that the clearance of these viruses does not appear to be mediated by virus-specific neutralizing antibodies and may rely on other antibody-mediated functions (antibody-dependent cellular cytotoxicity or phagocytosis) (Schuh et al., 2019). By contrast, upon experimental challenge, bats have been shown to generate virus-neutralizing antibodies to Nipah virus and rabies virus (Middleton et al., 2007; Turmelle et al., 2010). Concerning the immune cells present in bats, surprisingly, a high proportion of CD3+ T cells constitutively expressing mRNAs for IL-17A, IL-22 or Transforming Growth Factor Beta 1 (TGFB1) was detected. CD8+ T cells were also identified in the spleen as well as CD4+ T cells in the blood, lymph nodes and bone marrow and, to a lesser extent, B cells were detected in wild *P. alecto* (Martínez Gómez et al., 2016). The Major Histocompatibility Complex (MHC) molecules of bat, using *in vitro* experimental approaches, was shown to possess a unique consensus-binding motif and bind to larger peptides compared to other mammals. Finally, macrophages, monocytes and bone marrow derived DCs of bat were found to harbor phagocytic functions. Without a doubt, the long and complex history of co-evolution between bats and their viruses has played a crucial role in the construction of a bat-specific immune systems (Banerjee et al., 2019b, 2020; Letko et al., 2020). Undoubtedly, research on bats and the viruses they harbor, still requires a lot of effort. Many questions remain at the molecular and cellular levels, mainly because of to their great diversity, but also the lack of tools (*in vitro* and *in vivo* tools), the experimental limitations (e.g., limited repertoire of cell lines of different bat species, limitation in the isolation of bat viruses, propagation and virus stocks in bat cells instead of human cell lines) and the absence of experiments on live animals. It is particularly important to point out that all the current knowledge on the immune response in bats is highly fragmented and often obtained for a specific to a single pathogen in a single bat species or inferred from experiments conducted in limited cell models, so that no general conclusions should be made from any observation. The most revealing example is the primary cells of *P. alecto*, which have been described as constitutively expressing IFN-α mRNA, while it has never been found in the cells of *R. aegyptiacus*. Further studies remain necessary and essential to explore the complex relationship between bats and coronaviruses.

## Coronavirus Biology

### Coronavirus Structure

**FIGURE 3:**
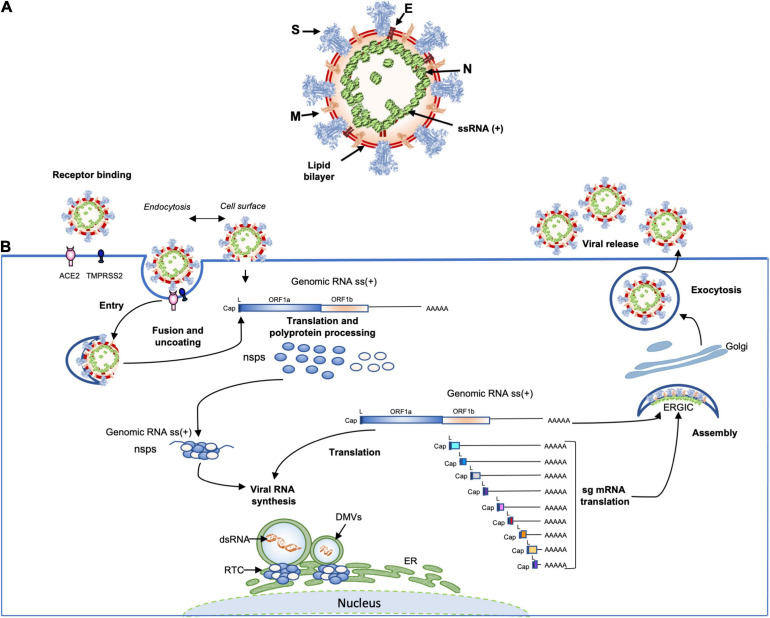
**(A)** Schematic representation of the SARS-CoV-2 virus structure. Together with membrane (M), envelope (E) transmembrane proteins, the spike (S) glycoprotein projects from a host cell-derived lipid bilayer. The positive-sense viral genomic RNA is associated with the nucleocapsid proteins forming the ribonucleoprotein (Mandala et al., 2020; Yao et al., 2020; Zhao et al., 2020). **(B)** The coronavirus life cycle. The coronavirus binds to the specific receptor (for SARS-CoV-2 the ACE2) together with the host factor TMPRSS2. Following entry, from the viral genomic RNA a translation of the two large open reading frames (ORF1a and ORF1b) occur. The resulting polyproteins are processed into individual non-structural proteins (16nsps) that form the replication and transcription complex (RTC). Formation of nuclear double membrane spherules (DMVs) associated with RTC allows viral genomic RNA replication and transcription of subgenomic mRNAs (sg mRNA). Produced structural proteins translocate into endoplasmic reticulum (ER) membranes and transit through the ER to the Golgi intermediate compartment (ERGIC) where nucleocapsid proteins (N) interact with newly produced genomic RNA resulting in the budding into the lumen of vesicular compartments. Finally, virions are secreted by exocytosis from the infected cell.

The coronavirus virions are spherical and ellipsoidal enveloped particles with an average diameter of 60–160 nm and a characteristic crown of spike proteins (Figure 3). The main structural components of coronaviruses are the spike (S), the envelop protein (E), the transmembrane protein (M) and the nucleoprotein (N) which forms a viral ribonucleoprotein (vRNP) complex with the viral RNA (vRNA). The first electron microscopy analysis on SARS-CoV-2 particles revealed that SARS-CoV-2 is consistent with these characteristics with diameters ranging from 53 to 110 nm including the contribution of the spikes (≅23 nm) (Klein et al., 2020; Yao et al., 2020; Zhou et al., 2020c). The *S* glycoprotein of SARS-COV-2, composed of S1 and S2 domains, which forms trimer (∼600-kDa), is heavily glycosylated with 66 N-linked glycans and found at average of 26 ± 15 distributed on each virion, half as much as SARS-CoV-1, which was estimated to possess an average of ∼50–100 S per virion (Neuman et al., 2011). The S1 glycoprotein trimer is the primary determinant of host interaction with three RBDs responsible for the binding of the virus to its receptor ACE2, while the S2 is involved in the membrane fusion process. An envelope protein (E) is also found on the surface of the virion of coronaviruses, it is the smallest of all the structural proteins (8–12 kDa). The E protein is an intrinsic membrane protein that has been described to be involved in a wide spectrum of functions. Indeed, the SARS-CoV-1 E protein has been found to be engaged in several steps of the viral life cycle, such as assembly, budding but also in pathogenesis by activating the host inflammasome (Schoeman and Fielding, 2019). The structure of the SARS-CoV-2 E protein (75 residues) has been solved by RMN and its influence on the capacity of the S protein to promote assembly of SARS-CoV-2 virus-like-particles was described (Boson et al., 2020; Mandala et al., 2020). The most abundant structural protein, the M multi-spanning membrane protein, defines the shape of the viral envelope, it is also the central organizer of coronaviruses assembly, interacting with all other major coronaviral structural proteins (Masters, 2006). During assembly of the virion, the M interacts with itself, with the nucleocapsid protein N, with E and with the S protein (Kuo and Masters, 2002; Boscarino et al., 2008; Arndt et al., 2010). SARS-CoV-2 M protein has recently been found to act as an antagonist of both types I and III IFNs by affecting the formation of the RIG-I/MDA-5–MAVS–TNF Receptor-Associated Factor 3 (TRAF3)–TANK-binding kinase 1 (TBK1) signalosome (Zheng et al., 2020). Moreover, the M protein was found to interact with the central adaptor protein MAVS impairing MAVS aggregation and recruitment of downstream TRAF3, TBK1, and IRF3, leading innate antiviral response attenuation. SARS-CoV-2 membrane glycoprotein M antagonizes the MAVS-mediated innate antiviral response (Fu et al., 2020). In the viral lumen, oligomers of N protein interact with the ∼30-kb-long single-stranded vRNA to form vRNPs (Kang et al., 2020). Until now, very little information on the structure of RNPs has been available. However, by studying the SARS-CoV-2 virus, the native conformation of vRNPs and their high order assembling were revealed for the first-time (Klein et al., 2020; Yao et al., 2020). An average of 38 vRNPs per virion was described. It has been proposed that vRNPs would be linked to other neighboring vRNPs as “beads on a string” in order to maintain a relative flexibility and allowing an efficient packaging of the very large 30 kb vRNA in the viral lumen of 80 nm diameter (Klein et al., 2020; Yao et al., 2020).

### Coronaviruses, Genome Organization and Viral Proteins

Coronaviruses have the largest RNA genomes (27–32 kb; SARS-CoV-2 reference sequence NC_045512: 29,903 kb) of any known virus family (Figure 1). The single-stranded positive sense vRNA genome contains a 5′ methyl-guanosine cap and a 3′ poly(A) tail. The order of genes is highly conserved among all coronaviruses. The genome comprises a basic set of genes, 5′-replicase-S-E-M-N-3′. The replicase gene includes two sub-units: ORF1a and ORF1b, that occupy two thirds of the viral genome and are translated into two polyproteins pp1a (nsp-1-11) and pp1ab (nsp-1-10, nsp-12-16) that are cleaved by viral proteases into 16 proteins involved in genome replication and in the subgenomic mRNAs (sg mRNAs) synthesis (Figure 3) (Yao et al., 2020). The two polyproteins are the only proteins translated from the genome whereas the products of all downstream ORFs are derived from sg mRNAs. Pp1a is cleaved into 11 nsps: nsp1 (19.6 kDa, 180 amino acids, host mRNA degradation, translation inhibition), nsp2 (70.5 kDa, 639 amino acids, unknown function), nsp3 (217 kDa, 1946 amino acids, polyprotein processing, de-ADP-ribosylation, deubiquitination, IFN antagonist), nsp4 [56 kDa, 501 amino acids, double membrane vesicles (DMV formation)], nsp5 (33.7 kDa, 307 amino acids, polyprotein processing, inhibition of IFN signaling), nsp6 (DMV formation), nsp7 (9.2 kDa, 84 amino acids, co-factor for RNA dependent RNA polymerase), nsp8 (21.8 kDa, 199 amino acids, primase or 3′ terminal adenylyltransferase, cofactor for RNA dependent RNA polymerase), nsp9 (12.3 kDa, 114 amino acids, RNA-binding protein), nsp10 (14.7 kDa, 140 amino acids, cofactor for nsp14 and 16), nsp11 (1.3 kDa, 13 amino acids, unknown function), whereas pp1ab is processed into nsp1-10, plus nsp12 (106.6 kDa, 932 amino acids RNA-dependent polymerase RNA polymerase, nucleotidyltransferase), nsp13 (66.8 kDa, 601 amino acids, helicase, RNA 5′ triphosphatase), nsp14 (59.8 kDa, 527 amino acids, 3′–5′ exonuclease, proofreading, RNA cap formation, guanosine N7-methyltransferase), nsp15 (38.8 kDa, 346 amino acids, endoribonuclease, evasion of immune response) and nsp16 (RNA-cap formation, ribose 2′-*O*-methyltransferase). The other one-third of the viral genome contains genes coding for virion structural proteins, S-E-M and N, described above. In addition to the basic genes downstream of the replicase gene, supplementary Open Reading Frames (ORFs), named “accessory” genes are interspersed between the main genes or embedded as alternative ORF within another gene [e.g., the internal (I) gene of MHV]. These “accessory” genes, appear to be specific within each coronavirus genus. They are labeled according to the smallest transcript in which they fall, so there is usually no relatedness. Some of these extra ORFs are thought to have been acquired through ancestral recombination with RNA from heterologous viral sources. Many of these genes are maintained in the genome of coronaviruses suggesting that they might play an important role in viral cycle or/and pathogenesis. The SARS-CoV-2 genome encodes at least for 9 non-structural ORFs: ORF3a, 3b 6, 7a, 7b, 8, 9b, 9c, and 10. Some of these ORFs are translated into accessory proteins: ORF3a, 6, 7a, 7b, 8 while others ORF3b and ORF10 might not be translated. These non-structural ORFs and the accessory proteins will be further described in more details.

## Coronavirus Replication Cycle

Coronavirus replication cycle is initiated by the binding of virions to cellular attachment factors and then to the specific interaction between S and the cellular receptor, such as ACE2 for SARS-CoV-2, together with host factor, such as TMPRSS2 which promotes viral uptake and fusion at the cellular membrane or endocytosed into endosomes where spike is processed by cathepsin L (Figure 3) (Murgolo et al., 2021). Following delivery and uncoating of the viral nucleocapsid to the cytoplasm, the translation of the two ORF1a and ORF1b from the genomic RNA start and, as we discussed above, two polyproteins are product (pp1a and pp1ab). The latter results from a programmed −1 ribosomal frameshift at the overlap of ORF1a et ORF1b (the coronavirus frameshifting stimulation element-stem-loop 1 of SARS-CoV-2, reference strain: NC_045512, is located between nucleotides 13,476–13,503) (Finkel et al., 2020). Thus, Pp1a and pp1ab are then processed by two cysteine proteases located within the nsp3 (papain-like protease) and nsp5 (chymotrypsin-like protease) respectively, into 16 mature products (nsp1 to nsp16). Rapidly after proteolytic cleavage, the nsp1 targets the host-cell translation (Schubert et al., 2020; Thoms et al., 2020), while the other processes nsps (nsp2-16) together with characteristic perinuclear double membrane vesicles (DMVs) that assemble to form a Replication and Transcription Complex (RTC) (Romano et al., 2020). The establishment of the RTCs is supposed to offer an optimal environment for RNA synthesis and prevent the exposure of viral replication intermediates to cytosolic innate immune sensors. However, a recent direct visualization of SARS-CoV-2 RTCs, using cryo-electron microscopy, revealed that the main component of RTCs interior is branched to double stranded RNA filaments and not proteins (Klein et al., 2020). This suggests that viral replicase complex is not located inside the RTCs, but instead, is associated with DMVs, and that viral RNAs intermediates accumulate into the RTC *via* a molecular pore complex newly described, interconnecting RTC interior and cytoplasm (Wolff et al., 2020). This viral replicase complex is based, on one hand, on nsp2-11 (factor assisting viral replication, host immune evasion or recruitment of intracellular membranes) and, on the other hand, on nsp12-16 that provide enzymatic functions, but also of several cellular proteins (V’kovski et al., 2019). Following the RTCs formation, the replication of genomic RNA and the transcription of multiple subgenomic mRNAs (1 h post-infection, the genome and sg mRNAs are detected) begin. The viral genomic replication starts by the synthesis of a full-length-negative-sense genomic copies that serve as template for the generation of new positive-sense genomic RNA. These new genomes are then packaged into new virions or used for translation to produce more nsps. During negative-strand RNA synthesis, a discontinuous transcription mechanism takes also place. The evidence for canonical discontinuous synthesis was first proposed following the discovery of a specific RNA sequence on the viral plus strand in addition to the poly(A) tract located at the 3′ end. Indeed, it has been shown that the genome and all sg mRNAs possessed, notably, at the end of the leader and before the body of each ORF, an “intergenic sequence” (IGS) or “transcription regulating sequence” [TRS, TRS-Body (TRS-B), TRS-Leader (TRS-L), which served to redirect the viral polymerase to an internal site and, then, served as a primer for elongation (van der Most et al., 1994; van Marle et al., 1995)]. During this event of (-) sg RNAs synthesis, when the TRS-body is reached, the transcription is interrupted and re-initiated at the TRS adjacent or switch to amplify the leader sequence (∼70 nt) at the 5′ end of the genome guided by complementarity of the TRS-B to the leader TRS (TRS-L). Following this process, a set of (-) sg RNAs is produced and used to synthetize a set of positive-sense sg mRNAs. For both human alpha-CoVs (HCoV-229E and HCoV-NL63), as well as the beta-CoVs (MHV, HCoV- OC43, and HCoV-HKU1), the TRS sequence is 5′ UCUAAAC3′, except for HCoV-OC43, where a C is replaced by a U (UCUAAAU). For SARS-CoV-1 and SARS-CoV-2, as well as bat SL-CoVs, the TRS sequence is 5′AAACGAAC3′. In addition to this canonical sg RNAs synthesis, a non-canonical sg RNAs production was recently described for SARS-CoV-2 and showed that, in addition to the genomic and 9 sg mRNAs, SARS-CoV-2 produces transcripts encoding unknown ORFs with fusion, deletion, and/or frameshift (Kim et al., 2020). Moreover, RNA modification sites on viral transcripts were also described, with the most frequent motif, AAGAA. The modification sites on the ‘AAGAA-like’ motif (including AAGAA and other A/G-rich sequences) are found throughout the viral genome but particularly enriched in genomic positions 28,500–29,500. Long viral transcripts (gRNA, S, 3a, E, and M) are more frequently modified than shorter RNAs (6, 7a, 7b, 8, and N), suggesting a modification mechanism that is specific for particular RNA species (Kim et al., 2020). Following transcription and translation, the structural proteins M, S, E, and N translocate into endoplasmic reticulum (ER) and transit through the ER-to-Golgi Intermediate Compartment (ERGIC) where N proteins interact with genomic RNA and condense with the M proteins, the envelope components to form virions, which then bud into lumen of secretory vesicular compartments. Finally, virions are secreted from the infected cells by exocytosis (V’kovski et al., 2020). As we have seen above, the biology of coronaviruses is complex and further studies on these viruses, their diversity and evolution are crucial today to better understand and anticipate other zoonotic events.

## Diversity of Coronaviruses

Coronaviruses can infect a variety of animals and humans. The intra- and interspecies transmissions of coronaviruses form a complex ecosystem promoting evolution and diversity. Previous studies on CoVs genomes have shown a higher composition in AU than in GC [selective advantage of the abundance of the A nucleotide (Kustin and Stern, 2021)]. For the SARS-CoV-2, in all the structural genes, either A or U nucleotides are the most predominant nucleotides and, in addition, A or U nucleotides are also the most predominant nucleotides at the 3rd position of codons implying a higher gene expression efficiency of these SARS−CoV−2 proteins (Kandeel et al., 2020; Sheikh et al., 2020). In term of evolution, two genes of SARS-CoV-2, M and E, tend to evolve slowly by accumulating nucleotide mutations while genes encoding N, viral replicase and S, tend to evolve faster (Dilucca et al., 2020). The diversity of coronaviruses can be explained at least by three main mechanisms (Anthony et al., 2017b). First, the error rate of the viral RNA polymerase. Indeed, in contrast to other RNA viruses that have high mutation rates (around 10^–4^ to 10^–6^ errors per nucleotide, which is equivalent to approximately one mutation per genome per replication cycle), coronaviruses replication fidelity is determined by the 3′-to-5′ exoribonuclease activity encoded in nsp14 that proofreads RNA during replication through excision of mismatched incorporated nucleotides. But mutations are still introduced (Eckerle et al., 2007, 2010; Ferron et al., 2018). For SARS-CoV-2, the genome mutation rate was evaluated at one or two mutations per month (Kupferschmidt, 2020). Second, adenosine-to-inosine and cytosine-to-uracil transitions can occur spontaneously through oxidative damage by free radicals or through the action of the Adenosine Deaminases that Act on RNA (ADAR) [that target double-stranded RNA (dsRNA) for deamination of adenines into inosines] and the Apolipoprotein B mRNA Editing Catalytic subunit (APOBEC) (that target single-stranded nucleic acids). These transitions, mostly occurring during the RNA plus strand replication and translation, cannot be corrected by the proof-reading machinery of coronaviruses. Thus, this leads to amino-acid (a.a) substitutions that can be quite drastic for the fitness of a virus or may help the virus to escape the immune response (Di Giorgio et al., 2020; Khrustalev et al., 2020; Ratcliff and Simmonds, 2021). Third, coronaviruses have the capacity to undergo both inter-molecular recombination between 2 distinct molecules and intra-molecular recombination within the same molecule. Thus, co-infection with related strains of MHV give birth to chimeric viral genomes by inter-molecular recombination (Makino et al., 1986; Keck et al., 1988). The civet SARS-CoV-1 strain-SZ3 arose through recombination of two existing bat strains, WIV16 and Rf4092 (Hu et al., 2017). As we discussed above, strong evidence also suggest that a recombination event occurred between HCoV-229E-like viruses found in *Hipposideros* bats and HCoV-NL63-like viruses found in *Triaenops afer* bats, where the S gene is more closely related to the HCoV-229E virus (Tao et al., 2017). Recombination is also implicated in the emergence of SARS-CoV-1 and MERS-CoV (Li et al., 2005; Hon et al., 2008; Lau et al., 2015a; Anthony et al., 2017a). In addition to inter-molecular recombination, intra-molecular recombination, at virus-specific transcription regulatory sequences (TRSs), to generate a set of sg mRNAs (sg mRNAs) with common 5′ and 3′ ends have been also observed (Dufour et al., 2011; Sola et al., 2015). The majority of recombination events, identified in coronaviruses isolated from bats, suggest recombination hotspots around the S gene (Hu et al., 2017). For the SARS-CoV-2, a study of genetic variation in patient samples has suggested that recombination may be occurring during infections in humans (Yi, 2020) and the most frequent recombination breakpoint have been located within the S gene, as previously found for other coronaviruses (Graham and Baric, 2010; Bobay et al., 2020; Tagliamonte et al., 2020; Zhu et al., 2020). In relation to this issue, at the whole genome level, the SARS-CoV-2, as discussed above, has been suspected to possibly originate from a recombination between bat and pangolin coronaviruses. Indeed, SARS-CoV-2 is 96.2% identical to bat-SL-CoV-RaGT13, however, the S region resembles to the divergent SARS strain isolated from Pangolin-CoV-2019 (Pangolin-CoV/hCoV-19/Pangolin/Guandong 2019). Indeed, the S gene of Pangolin-CoV-2019 shared 97.5% identity with S gene of SARS-CoV-2 while, at the whole genome level, the identity between SARS-CoV-2 and Pangolin-CoV-2019 showed lower sequence identity (91.02%) (Lam et al., 2020a; Wu et al., 2020; Xiao et al., 2020; Zhang et al., 2020a; Zhou et al., 2020c). Recently, Zhou et al. (2020b) discovered a new viral strain, RmYN02, from the bat *R. malayanus*, with 97.2% identity in the ORF1ab gene to SARS-CoV-2 (and only 61.3% similarity to in the RBD motif), which harbors multiple a.a insertions at the S1/S2 cleavage site in the S protein. Altogether, CoVs mutations and recombination can offer a rapid means of acquiring gene variants that facilitate host switching, but also induce vaccine failure, as it has been already previously observed (Chen et al., 2017; Feng et al., 2018). The adaptive capacity of CoVs largely results from their large genomes, which reduce the risk of deleterious mutational errors and facilitate recombination events. Large CoV genomes are made possible by the unique proofreading capacity encoded for their RNA-dependent polymerase. Overall, the study of recombination events and mutation processes are a key consideration in the context of the current SARS-CoV-2 pandemic as well as for future animal and zoonotic coronaviruses, especially considering the acquisition of accessory genes.

## Coronavirus Accessory Proteins

Coronavirus accessory proteins have been less studied than other proteins of the virus for two main reasons (Figure 1). First, they are often dispensable for *in vitro* viral replication, but play a crucial role in pathogenesis and virus fitness under the natural environment of the host (Narayanan et al., 2008; Menachery et al., 2017). Second, bioinformatics approaches to detect accessory proteins are generally strong challenges and particularly for overlapping genes, which encode for accessory proteins that are often overlooked during genome annotation (Pavesi et al., 2018). Accessory genes are present in greater or lesser number in various CoVs and their functions are still incompletely understood. One of the main characteristics of the SARS-CoV-1, MERS-CoV, SARS-CoV-2, and bat SL-CoVs is the large number of accessory genes in comparison to other coronaviruses. For example, the SARS-CoV-1 retains 9 ORFs while the HCoV-HUK1 contains only three accessory ORFs (ORF4, ORF8 and one putative ORFI). The origin of these accessory genes, remains an open question as most of them have no identifiable orthologs in protein databases. It is possible that some of them have evolved through intragenomic recombination, as suggested for several of the accessory genes of SARS-CoV-1, but it is also probable that some of these extra ORFs have been acquired through ancestral recombination with RNA from cellular or heterologous viral sources. One of the best examples of this possible horizontal gene transfer is the hemagglutinin esterase (HE) gene, which is homologous to an influenza C virus gene (Luytjes et al., 1988; Zeng et al., 2008). Other candidates have also been depicted: the 2a gene found in MHV, HCoV-OC43, but also MERS-CoV (named ORF4b in MERS-CoV), which encodes a 2′5′ phosphodiesterase that antagonizes IFN (Goldstein and Weiss, 2017), but also the ORF6 and the ORF10 of beluga whale coronavirus that encoded proteins possessing a.a similarity to human astrovirus capsid proteins and uridine kinase that, until now, has never been described in viruses (Mihindukulasuriya et al., 2008). Mutational knockdown or deletion of accessory genes has revealed, as described above, that none of them are essential for viral replication *per se*, however, deletion of some of these genes can have a profound effect on viral pathogenesis, including impacts on host innate immunity (de Haan et al., 2002). Indeed, the deletion of the gene 7 of TGEV was shown to accelerate growth kinetics and pathology (Cruz et al., 2011). This observation was also reinforced when the ORF6 of SARS-CoV-1 was transfer to a related mouse virus. The replication of the recombinant MHV was enhanced (Tangudu et al., 2007).

The SARS-CoV-1 genome possesses 9 ORFs coding for accessory proteins in the 3′-end of its genome. Two are located between the S and the E genes (ORFs 3a, 3b), five between the M and the N genes (6, 7a, 7b, 8a, and 8b) and two within the N gene (ORF9b and a putative ORF9c). Although some of these genes are presents among human and some animal coronaviruses, no high similarities with accessory genes of other coronaviruses belonging to other genera was found (Lai and Cavanagh, 1997). Since 2003, the field of coronavirus accessory proteins has gained significant attention and reverse genetics approaches gave some clues concerning the role of some of them. For the SARS-CoV-2, a controversy still persists. Indeed, some reports described 11 predicted accessory protein ORFs (ORF3a, 3b, 3c, 3d, 6, 7a, 7b, 8, 9b, 9c, and 10) while others concluded that the SARS-CoV-2 express only ORF3a, 6, 7a, 7b, 8 or more or less ORF3b, 3c, 9b, 9c, and 10 (Gordon et al., 2020; Jungreis et al., 2020; Kim et al., 2020; Michel et al., 2020).

### Accessory Proteins of SARS-CoV-1 and SARS-CoV-2

Previous studies have been shown that deletion of six of the eight ORFs of SARS-CoV-1 (ORF3a, ORF3b, ORF6, ORF7a, ORF7b, and ORF9b), alone or in combination, does not dramatically influence the level of RNA or the replication efficiency of SARS-CoV-1 *in vitro* or *in vivo* in a mouse model (Yount et al., 2005; von Brunn et al., 2007). Although most of the accessory proteins do not appear to play a central role in viral replication, their functions in the virus-host interaction and pathogenicity are determinant.

### ORF3a and 3b Proteins

**FIGURE 4:**
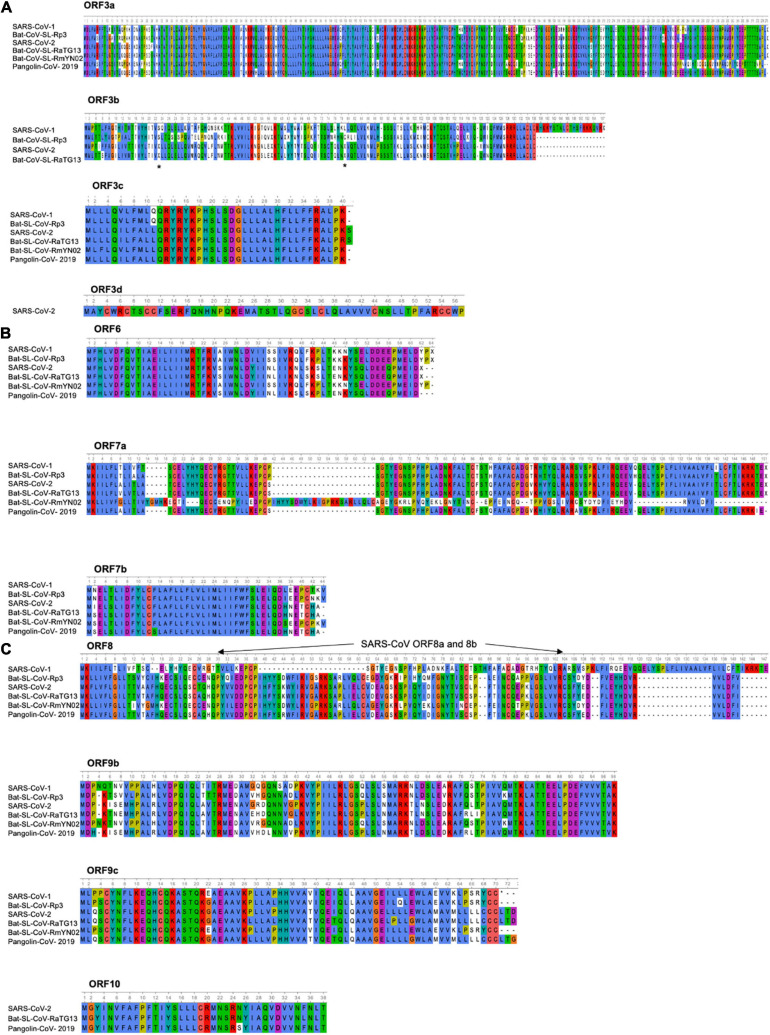
Amino acid alignment of ORFs encoding accessory proteins. **(A)** Amino acid alignment of ORF3a and 3b sequences of SARS-CoV-1 (AY274119), Bat-SL-CoV-Rp3 (DQ071615), SARS-CoV-2 (NC_045512), Bat-SL-CoV-RaTG13 (MN996532), Bat-SL-CoV-RmYN02 (JX993988), Pangolin-CoV-2019 (MT121216). No ORF3b is found in the Bat-SL-CoV-RmYN02 and Pangolin-CoV-2019 sequences, the stars indicate the stop codons. Amino acid alignment of ORF3c and 3d sequences. ORF3d is only found in the SARS-CoV-2. The asterisks indicate the stop codons. **(B)** Amino acid alignment of ORF6, 7a and 7b sequences. **(C)** Amino acid alignment of ORF8, 9b, 9c, and 10 sequences. The SARS-CoV-1 ORF8 went through a gradual deletion over the course of the epidemic and at the end of the outbreak ORF8 was divided in 2 ORFS (ORF8a and 8b). ORF10 is only found in SARS-CoV-2, Bat-SL-CoV-RaTG13 and Pangolin-CoV-2019. Sequences were analyzed using Unipro UGENE: a unified bioinformatics tollkit Okonechnikov; Golosova; Fursov. Bioinformatics 2012 28: 1,166–1,167. Amino-acids are color-coded according Clustal X. For each ORF, the SARS-CoV-2 sequence (NC_045512) was used as a reference sequence to perform the alignment.

The SARS-CoV-1 encodes ORF3a and ORF3b proteins that can be both detected in SARS-CoV-1-infected tissues (Chan et al., 2005). The ORF3a of SARS-CoV-1 is an *O*-glycosylated 274 a.a long protein that contains three transmembrane domains and, in the C-terminus part, two intracellular protein sorting and trafficking signals (YXX and di-acidic motifs), which are important for the transport of ORF3a protein to the cell surface (Figure 4A and Supplementary Table 1). Multiple functions have been assigned to the ORF3a protein. As the ORF3a protein was shown to form a homo-tetramer complex, hypothesis have been made about a possible formation of a potassium-permeable channel-like structure. In addition of this function, the ORF3a protein was found to interact with the structural proteins, S, E, M, and the ORF7a in the Golgi apparatus proximal to the site of virus assembly and budding (Tan et al., 2004b; Zeng et al., 2004; Yuan et al., 2005a). Two studies demonstrated that the ORF3a protein is a *bona fide* viral structural protein (Ito et al., 2005; Shen et al., 2005), and that, although it is incorporated into virus-like particles (VLPs), it is not necessary for the particles formation. *In vitro* expression studies revealed that the ORF3a protein induces, G1 phase cell cycle arrest by reducing cyclin D3 expression and inhibiting retinoblastoma protein (Rb) phosphorylation (Yuan et al., 2007), and also apoptosis in Vero E6 cells (Law et al., 2005b). Moreover, electron microscopy analyses showed that the ORF3a protein induced Golgi fragmentation and an accumulation of intracellular vesicles (Freundt et al., 2010). In addition to an intracellular effect, the ORF3a protein was also described to be released from protein expressing cells (Huang et al., 2006b). Although the biological role of this release remains unclear, some observations about the presence of a strong, and maybe protective, humoral response against this protein in SARS-CoV-1 patients, suggest that this ORF protein is playing an important role in viral pathogenesis (Zhong et al., 2006). Indeed, the immunization of rabbits with a synthetic peptide corresponding to the N amino terminal domain of the ORF3a protein results in the induction of neutralizing antibodies that inhibit SARS-CoV-1 infection in VeroE6 cells. Finally, the ORF3a protein of SARS-CoV-1 was also shown to activate NF-kB, a critical transcription factor involved in the activation of pro-inflammatory genes (Kanzawa et al., 2006), and the NLRP3 inflammasome by promoting TRAF3-dependent ubiquitination of adaptor protein apoptosis-associated speck-like protein containing a caspase recruitment domain (Siu et al., 2019). The SARS-CoV-2 ORF3a protein, which presents 72.4% a.a identity with the SARS-CoV-1 ORF3a protein and 97.8% with the bat-SL-CoV-RaTG13 ORF3a, retains six functional domains (I–VI) (Figure 4A and Supplementary Table 1). These functional domains are linked to virulence, infectivity, ion channel formation and virus release (Issa et al., 2020; Kern et al., 2020) and as the ORF3a protein of SARS-CoV-1, the ORF3a protein SARS-CoV-2 retains the capacity to induce apoptosis (Ren et al., 2020) and also to inhibit fusion autophagosomes with lysosomes (Zhang et al., 2021b).

The ORF3b of SARS-CoV-1 encodes for a protein (154 a.a) that has been shown to be localized to the nucleolus and/or mitochondria (Yuan et al., 2005b, 2006a; Kopecky-Bromberg et al., 2007). *In vitro* expression studies showed that the ORF3b protein of SARS-CoV-1 induces a cell growth arrest in G0/G1 phase (Yuan et al., 2005b) and can stimulate both apoptosis and necrosis in Vero E6 cells (Khan et al., 2006). The ORF3b protein of SARS-CoV-1 was described to possess, in its C-terminal part, a nuclear localization signal (a.a 134–154). Interestingly, the ORF3b homologs identified from three bat-SL-CoV strains (Bat-SL-CoV-Rf1, -Rm1 and -Rp3) were C-terminally truncated, lacked the C-terminal NLS of SARS-CoV-1 and display a different cellular localization (ORF3b of bat-SL-CoV-Rf1, -Rm1 and -Rp3 were only found in cytoplasm). The ORF3b of SARS-CoV-1 and bat-SL-CoV (Bat-SL-CoV-Rm1) were proposed to antagonize IFN by modulating the activity of IRF3 (Zhou et al., 2012), while the ORF3b protein of bat-SL-CoV-Rp3 showed no IFN antagonism. This observation is particularly important. Indeed, it is tempting to speculate that the ORF3b derived from bat SL-CoVs could have a different IFN antagonism profiles in bat cells just like the ORF3b of SARS-CoV-1. Recently, it was described the presence of a premature stop codon in the ORF3b gene of SARS-CoV-2 (Figure 4A and Supplementary Table 1). Furthermore, SARS-CoV-2 natural variants, isolated from two patients, in which a longer ORF3b reading frame was shown to be associated with severe disease and increased ability to suppress IFN response (Konno et al., 2020). As describe (Figure 4A and Supplementary Table 1), the ORF3b of SARS-CoV-2 has a high identity percentage (93.3%) with the ORF3b of the bat-SL-CoV-RaTG13 and does not contain the C-terminal part (which encompass a NLS). However, stop codons are retained in both sequences (Figure 4A).

Finally, some additional ORFs have been proposed for the SARS-CoV-2: ORF3c and ORF3d (Figure 4A and Supplementary Table 1). Indeed, a putative ORF, named ORF3c was proposed to overlap the ORF3a in an alternative reading frame and, recently, a ribosome profiling study confirmed that the ORF3c is indeed translated during infection. Moreover, the ORF3c (40–41 a.a predicted transmembrane protein) was found to be conserved across the subgenus *Sarbecovirus* (Firth, 2020). The ORF3d was also detected in Guangxi pangolin-CoVs and SARS-CoV-2, but not in other closely related pangolin-CoVs or bat-SL-CoVs (Chan et al., 2020; Nelson et al., 2020; Pavesi, 2020). To note, the ORF3d has been mistaken with the previously documented ORF3b in several studies (Fung et al., 2020; Ge et al., 2020; Hachim et al., 2020; Helmy et al., 2020; Yi et al., 2020; Jungreis et al., 2021).

### ORF6 Protein

The SARS-CoV-1 ORF6 protein is a 63 a.a, membrane associated protein (Figure 4B and Supplementary Table 1). Its expression was detected in virus-infected Vero E6 cells as well as in the lung and intestine tissues of SARS-CoV-1 patients (Geng et al., 2005; Pewe et al., 2005). The ORF6 protein was described to mainly localized in the ER and Golgi compartments in virus-infected cells, but was also found to be incorporated into viral particles and released (Huang et al., 2007). In studies using recombinant MHV, the ORF6 protein of SARS-CoV-1 was shown to be associated with viral RNA, co-localized with replicating viral RNA on cytoplasmic vesicles and enhanced MHV viral RNA and protein synthesis (Frieman et al., 2007). Furthermore, studies showing the interaction of the ORF6 protein with the nsp8 support the notion that the ORF6 protein could play a role in virus replication (Tangudu et al., 2007). Several studies revealed that the ORF6 protein blocked IFN-induced STAT1 nuclear translocation by retention of the nuclear import-adaptor-molecule karyopherin alpha 2 in the cytoplasm (Kumar et al., 2007). Finally, the ORF6 protein of SARS-CoV-1 was shown to be required for optimal replication. As for the ORF3a and ORF3b proteins, the ORF6 protein of SARS-CoV-2 presented a strong similarity with the ORF6 protein of SARS-CoV-1 and bat-CoV-SL-RmYN02 (68.9 and 70.5%, respectively), but even more with the ORF6 protein of the bat-SL-CoV-RaTG13 and Pangolin-CoV-2019 (100 and 96.7%) (Figure 4B and Supplementary Table 1). Recently, SARS-CoV-2 ORF6 deletion variants were isolated and characterized (26-nt deletion and 34-nt deletion, respectively) (Quéromès et al., 2021) or carrying a nucleotide mutation leading to a stop codon in the ORF6 protein (Delbue et al., 2021). No significant difference in term of viral replication, as well as ISG expression, was observed between these two SARS-CoV-2 deletion variants and the reference strain. However, an upregulation of several genes, all involved in the NF-kB pathway, was noted after infection with these variants in comparison to the reference strain, suggesting that the truncated ORF6 proteins could play a role in the inflammatory host-response (Xia et al., 2020). Lately, the SARS-CoV-2 ORF6 protein function as a potent IFN antagonist (Yuen et al., 2020; Kimura et al., 2021) and also was found to block nuclear import of a wide range of host factors through interactions with RAE1 and NUP98 (Addetia et al., 2021).

### ORF 7a and ORF 7b Proteins

Both SARS-CoV-1 ORF7a and ORF7b proteins have been shown to be dispensable for virus replication (Figure 4B). The 122 a.a SARS-CoV-1 ORF7a protein is a type I transmembrane protein containing a signal peptide sequence (15 amino-acid), an 81 a.a lumenal domain, a 21 a.a transmembrane domain and a short C-terminal tail (Figure 4B). The ORF7a protein was found to be present in the perinuclear region in SARS-CoV-1-infected cells, in the ER or the ERGIC (Sims et al., 2005; Yount et al., 2005), the *trans*-Golgi network (Fielding et al., 2004; Nelson et al., 2005) and also to be incorporated into purified SARS-CoV-1 particles (Huang et al., 2006a). Several biological functions have been assigned to the SARS-CoV-1 ORF7a protein. These include the induction of apoptosis through a caspase-dependent pathway (Tan et al., 2004a; Schaecher et al., 2007), the inhibition of cellular protein synthesis, the activation of p38 mitogen-activated protein kinase (Kopecky-Bromberg et al., 2006) and the cell cycle arrest at the G0/G1 phase (Yuan et al., 2006b). The ORF7a protein was also shown to enhance pro-inflammatory cytokine production through the activation of NF-kB and JNK in A549 cells (Kanzawa et al., 2006). Finally, the ORF7a protein was found to inhibit BST-2 glycosylation, leading to a loss of its antiviral function (Kanzawa et al., 2006; Taylor et al., 2015). The detection of anti-ORF7a antibody in SARS-CoV-1 patient serum suggests that it is expressed in infected patients (Guo et al., 2004).

For the SARS-CoV-2, the ORF7a protein has been found to interacts with the ribosomal transport proteins HEATDR3 and the AAA-ATPase Midasin-1 (MDN1) and to antagonize type I IFN signaling (Gordon et al., 2020; Xia et al., 2020). Moreover, the SARS-CoV-2 ORF7a protein was depicted to be an immunomodulating factor for immune cell binding (HLA-DR/DP/DQ) and to trigger dramatic inflammatory responses (upregulation of proinflammatory cytokines, including IL-6, IL-1β, IL-8, and 38 TNF-α) (Zhou et al., 2021). A deletion of 27 a.a, which maps to a putative signal peptide within the ORF7a protein, has recently been reported in two SARS-CoV-2 isolates from two separate patients (Holland et al., 2020). This large deletion of the ORF7a is proposed to prevent redundant functions on inhibition of cellular protein synthesis also found associated with the ORF6 protein that, as we have seen above, interacts with the mRNA export proteins NUP98 and RAE1 and may inhibit cellular translation (Addetia et al., 2020, 2021). The a.a sequence of the SARS-COV-2 ORF7a protein is very close to the ORF7a of bat-SL-CoV-RaTG13 ORF7a like that of the Pangolin-CoV-2019, but much more distant than that of the bat-SL-CoV-RmYN02 (97.5,97.5, and 40% a.a identity, respectively).

The ORF7b of SARS-CoV-1 was predicted to encode an extremely hydrophobic 44 a.a protein (Figure 4B) (Pekosz et al., 2006). During the outbreak of SARS-CoV-1 in 2002–2003, a virus was isolated from a patient in Frankfurt (strain Frankfurt-1). Frankfurt-1 isolate was described to possess a 45-nucleotide deletion in the transmembrane domain of the ORF7b protein (Pfefferle et al., 2009a). Interestingly, when expressed in HEK 293T cells, the full-length protein, but not the ORF7b with the deletion caused IFN-β induction and cleavage of procaspase 3. In CaCo-2 and HUH7 cells, but not in Vero cells, the Frankfurt-1 carrying the ORF7b deletion was shown to present a replicative advantage in regard to the virus containing an intact ORF7b. This effect was neither associated with changes in the induction or secretion of type I IFN, nor with altered induction of apoptosis in cell culture. However, pretreatment of cells with IFN-β caused the deleted virus to replicate to higher titers than the parental strain (Pfefferle et al., 2009a).

The ORF7b protein of SARS-CoV-2 presents a strong similarity with the ORF7b of bat-SL-CoV-RaTG13 and Pangolin-CoV-2019 (97.7 and 95.3% a.a identity, respectively) while some a.a variations are observed in the C-terminal part of the SARS-CoV-1 ORF7b protein of, bat-CoV-SL-Rp3 and bat-CoV-SL-RmYN02 (85.4, 85.7, and 85.4% a.a identity, respectively) (Figure 4B and Supplementary Table 1). The ORF7b protein of SARS-CoV-2 has been shown to inhibit type I IFN signaling by inhibiting the STAT1 phosphorylation (Xia et al., 2020).

### ORF8 Proteins of SARS-CoV-1 and SARS-CoV-2

During the outbreak of SARS-CoV-1 in 2002–2003, the coding sequence of SARS-CoV-1 ORF8 (122 a.a) went through a gradual deletion over the course of the epidemic and most of the human isolates of SARS-CoV-1 presented a naturally deletion in ORF8 (deletion of 29 nucleotides) at the end of the outbreak, resulting in two ORFs: ORF8a (39 a.a) and ORF8b (84 a.a) (Figure 4C). The expression of the ORF8a and ORF8b proteins, produced from SARS-CoV-1 sg RNA 8, was later confirmed in infected cells and insertion of the 29 missing nucleotides into human isolates, which merges ORF8a and 8b into a continuous ORF8, showed little impact on virus growth and on RNA replication, suggesting that the deletion might not be responsible for the increased pathogenicity (Keng et al., 2006). However, lately, replication in primate cell lines, as cell line generated from the lung of a *rhinolophid* bat as well as human airway epithelial cultures, revealed that the 29nucleotides deletion conferred an attenuation of replication level (Yount et al., 2005). Less frequent deletion, which led to large deletions (82 nt or 415 nt) or complete loss of ORF8, were also observed (Muth et al., 2018). In the expression studies, the SARS-CoV-1 ORF8a protein was shown to interact with the S protein, while the ORF8b protein interacts with M, E, ORF3a and ORF7a proteins. The SARS-CoV-1 ORF8a protein was also proposed to form an ion channel and the ORF8b protein was showed to down-regulated the E protein level (Chen et al., 2011). Finally, recently, the aggregation of the ORF8b proteins of SARS-CoV-1 was shown to induce ER stress, lysosomal damage, and subsequent activation of autophagy and lysosome machinery (Keng et al., 2006; Shi et al., 2019).

For the SARS-CoV-2, the protein encodes by this ORF8 is similar to the ORF8 protein of bat-SL-CoVs (Bat-CoV-SL-RaTG13 and Pangolin-CoV-2019: 95% a.a identity for both sequences), but presents a low homology with the SARS-CoV-1, bat-CoV-SL-RmYN02 and bat-SL-CoV-Rp3 ORF8 protein (26.2, 57.9, and 56.2%) (Figure 4C and Supplementary Table 1). The ORF8 protein of SARS-CoV-2 has been shown to strongly inhibit the IFN-stimulated response element (ISRE) after IFN-β treatment. The crystallization of SARS-CoV-2 ORF8 revealed a structure similar to the one of SARS-CoV-1 ORF7a, with the addition of two dimerization interfaces unique to SARS-CoV-2 ORF8 (Li et al., 2020, p. 8). In contrast to the SARS-CoV-1, the SARS-CoV-2 isolates presenting a large deletion that truncates the ORF7b and ORF8 (382-nt deletion) was showed to induce a significantly higher replication level than the wild type (Flower et al., 2020), but less severe infection and lower concentrations of proinflammatory cytokines, chemokines, and growth factors (Young et al., 2020). Recently, the ORF8 protein was shown to contributes to cytokine storm during SARS-CoV-2 infection by activating IL-17 pathway (Lin et al., 2021).

### ORF9b and 9c Proteins

**FIGURE 5:**
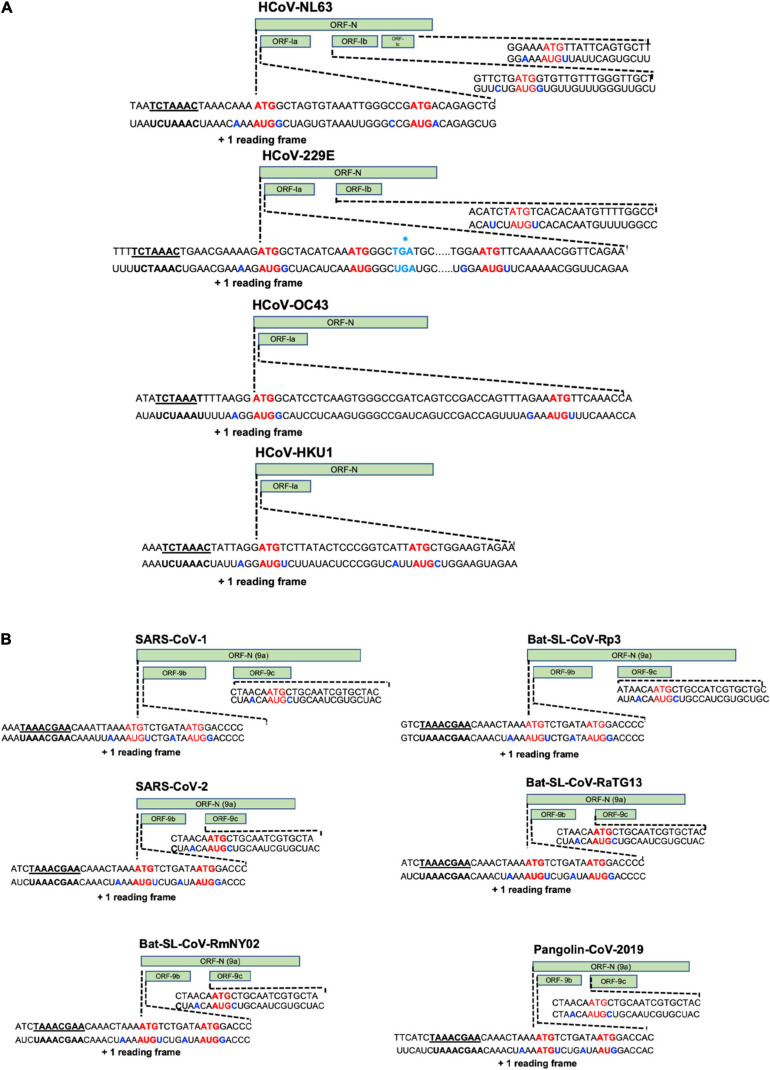
Expression of the ORF accessory genes embedded in the N gene. **(A)** AlphaCoV. HCoV-NL63 (AY567487), the initiation of the ORFN represented an optimal Kozak context (A at –3, G at +4). The initiation of the ORFIa indicated a suboptimal Kozak context (C at –3, A at +4). The initiation of the ORFIb represented a suboptimal Kozak context (C at –3, G at +4) (281). The initiation of the ORFIc indicated a suboptimal Kozak context (A at –3, U at +4). HCoV-229E (AF304460), the initiation of the ORFN represented an optimal Kozak context (A at –3, G at +4). The initiation of the ORFIa indicated a suboptimal Kozak context (G at –3, U at +4). Even if a start codon is presented at the same site as the start codon of ORF9b of SARS-CoV-1 or SARS-CoV-2, a stop codon (asterisk) is found on the third codon following the start codon. The initiation of the ORFIb represented a suboptimal Kozak context (U at –3, U at +4). BetaCoVs. HCoV-OC43 (AY903460), the initiation of the ORFN represented an optimal Kozak context (A at –3, G at +4). The initiation of the ORFI indicated a suboptimal Kozak context (A at –3, U at +4). HCoV-HKU1 (AY597011), the initiation of the ORFN represented a suboptimal Kozak context (A at –3, U at +4). The initiation of the ORFI indicated a suboptimal Kozak context (A at –3, C at +4). **(B)** BetaCoVs. SARS-CoV-1 and Bat-SL-CoV-Rp3, the initiation of the ORFN represented a suboptimal Kozak context (A at –3, U at +4). The initiation of the ORF9b indicated an optimal Kozak context (A at –3, G at +4). The start codon for ORF9b is very close to the start codon for ORFN (only 10 nucleotides between the ATG of N and the ATG of OR9b). The initiation of the ORF9c represented a suboptimal Kozak context (A at –3, C at +4). SARS-CoV-2, Bat-SL-CoV-RaTG13, Bat-SL-CoV-RmYN02 and Pangolin-CoV-2019, the initiation of the ORFN represented a suboptimal Kozak context (A at –3, U at +4). The initiation of the ORF9b indicated an optimal Kozak context (A at –3, G at +4). The start codon for ORF9b is very close to the start codon for ORFN (only 10 nucleotides between the ATG of N and the ATG of OR9b). The initiation of the ORF9c represented a suboptimal Kozak context (A at –3, C at +4).

Overlapping reading frames have been discovered in many organisms, but they are a common feature in viruses (Pavesi et al., 2018). In this context, the same nucleotide sequence codes for more than one protein in a different reading frame. These overlaps are typically assumed to be a form of genome compression, allowing the virus to increase its repertoire of proteins without increasing its genome length (Pavesi et al., 2018; Pavesi, 2019). Proteins created by gene overlaps (sometimes called “overprinting” or “overprinted,” or “novel”) are typically accessory proteins that play a role in viral pathogenicity or spreading (Rancurel et al., 2009; Pavesi, 2019). Several alpha-CoVs (as HCoV-229E and HCoV-NL63), but also beta-CoVs (as MHV, BCoV, HCoV-OC43, and HCoV-HKU1) have one or several alternative ORFs (from one to three) embedded entirely within their nucleocapsid gene (N). In the beta-CoVs genus, there is a relative sequence conservation of one ORF named “I” in the two coronaviruses MHV and BCoV (44.1% a.a identity), but a very low similarity is detected with the SARS-CoV-1 or SARS-CoV-2 ORF-9b (Supplementary Table 2). Functionally, the ORF-9b homolog, named “I,” in the +1 reading frame with respect to the N gene, of MHV was shown to act as an accessory structural protein that is not essential for viral infection, but confers a growth advantage (Fischer et al., 1997). Endemic human alpha-CoVs (HCoV-229E and HCoV-NL63) and human beta-CoVs (HCoV-OC43 and HCoV-HKU1) also present alternative ORFs embedded entirely within their nucleocapsid gene (N), but again a very low similarity with the ORFs Ia or I of HCoV-229E, HCoV-NL63, HCoV-OC43, HCoV-HKU1 was observed with the ORF-9b SARS-CoV-1 or SARS-CoV-2 (Supplementary Table 2). The SARS-CoV-1 ORF9b protein (98 a.a residues, Figure 4C and Supplementary Table 1) has been shown to be expressed in infected cells, from an internal ORF in the N gene by a leaky ribosomal scanning (Xu et al., 2009), but was also identified in the intestinal surface enterocytes and pneumocytes (Chan et al., 2005). Viruses are known to operate with several non-canonical translational mechanisms (re-initiation, ribosomal shunt, internal initiation, and leaky scanning), which may be programmed or incidental. The latter can be considered as translation noise, not subject to strong purifying selection, and therefore generally not phylogenetically preserved. However, in some cases, programmed exceptions tend to be subjected to purifying selection and phylogenetic preservation. Leaky scanning is the most common process used by RNA viruses to facilitate the access to ORFs. In this mechanism, a significant proportion of scanning ribosomes fail to initiate at the first AUG codon (suboptimal context surrounding the first AUG codon). In mammals, the optimal context for recognition of the AUG Start codon is GCCRCCAUGG, including a purine (R) at position −3 and a G at position +4 highly conserved and is especially important in the absence of an A at position −3 (Kozak, 1986, 2002) but instead, continue scanning until they reach an alternative initiation codon further downstream (Figure 5A). Leaky scanning is employed by numerous viruses (plants viruses and mammalian viruses). Besides suboptimal context surrounding the first AUG codon, leaky scanning may also be promoted by a number of other mechanisms. If an AUG codon is very close to the 5′ end of the transcript, then it is often not recognized efficiently (Sedman et al., 1990; Kozak, 1991). The close proximity of a downstream AUG codon to a preceding AUG codon (e.g., within approximatively 10 nt) can also increase the efficiency of leaky scanning (Williams and Lamb, 1989; Matsuda and Dreher, 2006). Leaky scanning can also be promoted by short upstream ORFs. In SARS-CoV-1, as well as SARS-COV-2, bat-CoV-SL-Rp3, -RaTG13, -RmNY02 and Pangolin-CoV-2019, the start codon for ORF9b is very close to the start codon for ORFN, with only 10 nucleotides in between (Figure 5B). Antibodies against the ORF9b protein were detected in the sera of SARS-CoV-1 patients demonstrating that this protein is produced during infection (Chow et al., 2006). The expression level of the SARS-CoV-1 ORF9b was shown to be relatively weaker when compared to that of the N protein (Xu et al., 2009) and to be dispensable for virus replication *in vitro.* The crystal structure of the SARS-CoV-1 ORF9b protein was solved in 2006 and revealed a novel dimeric tent-like β structure with an amphipathic surface and a central hydrophobic cavity, which binds lipid molecules that probably allows its association with intracellular vesicles (Meier et al., 2006). This hypothesis was reinforced by the observation, in mammalian cells, of the SARS-CoV-1 ORF-9b protein distributed diffusely within cytoplasm and nucleus (HeLa cells) (von Brunn et al., 2007) or associated with intracellular vesicles (HEK 293T cells) (Meier et al., 2006). Intraviral protein interactions study, using a yeast-two-hybrid approach, revealed that ORF9b protein is the most dominant interactor of the accessory proteins (The ORF9b protein showed interactions with at least 10 nsps, the E protein and four accessory proteins: the ORF8a, ORF8b, ORF9b, and ORF9c) (von Brunn et al., 2007). Currently, there is no explanation for this large number of interactions and the precise function of the SARS-CoV-1 ORF9b protein is still not understood, although it has been proposed that the ORF9b protein might contribute to virus assembly as a membrane-attachment point for other viral proteins, like N protein (Meier et al., 2006). As some other ORF proteins, the SARS-CoV-1 ORF9b protein was found to be a virion-associated protein (Xu et al., 2009). More recently, it has been shown that the SARS-CoV-1 ORF9b protein limits host cell IFN responses by targeting several proteins of the MAVS signalosome to degradation. Finally, transient ORF-9b expression was showed to result in a strong induction of autophagy (Shi et al., 2014). Another overlapping gene was proposed in the SARS-CoV-1 genome, the ORF9c. It has been reported within the nucleocapsid gene and coding for a predicted protein of 70 a.a (Marra et al., 2003; Rota et al., 2003). Until now, the SARS-CoV-1 ORF9c expression has not been demonstrated (Figure 4C and Supplementary Table 1). Since the SARS-CoV-2 outbreak, Ribo-seq and sequencing to systematically delineate the landscape of translated ORFs of SARS-CoV-2 and their expression during infection were done (Gordon et al., 2020; Kim et al., 2020). RNA-seq and ribosome profiling analyses of SARS-CoV-2 infected Vero E6 cells revealed out of frame internal initiations within ORFN (ORF9b and ORF9c) (Finkel et al., 2020) supporting the conclusion that these two SARS-CoV-2 proteins are expressed in SARS-CoV-2-infected cells. In addition, Gordon et al. (2020) demonstrated that stable expression of the SARS-CoV-2 ORF9b and ORF9c can occur in transfected HEK 293T and interactome studies showed that the ORFs proteins interact with several proteins as the mitochondrial import receptor subunit TOM70 (TOMM70) and the mitochondrial chaperone BCS1 (BCS1L), respectively. Finally, transcriptomic and proteomic studies, revealed that the ORF9b transcript and ORF9b protein (97 a.a) are detected in of SARS-CoV-2-infected cells (Bojkova et al., 2020; Nomburg et al., 2020) supporting, one more time, the notion that ORF9b is a *bona fide* SARS-CoV-2 protein. As for the SARS-CoV-1, antibodies against SARS-CoV-2 ORF9b were detected in the sera of convalescent patients (Personal communication) (Guo et al., 2004; Qiu et al., 2005; Jiang et al., 2020a). The SARS-CoV-2 ORF9b protein was depicted to inhibit the type I IFN response through its interaction with the outer membrane mitochondrial adaptor TOM70 (Jiang et al., 2020b). Recently, the crystal structure of ORF9b in complex with the human TOM70 was resolved (Gao et al., 2021). Moreover, SARS-CoV-2 ORF9b antagonizes types I and III interferons by targeting multiple components of the RIG-I/MDA-5-MAVS, TLR3-TRIF, and cGAS-STING signaling pathways (Han et al., 2021). As others ORFs, the SARS-CoV-2 ORF9b protein was found to be very similar to the ORF9b of bat-CoV-RaTG13 and the Pangolin-CoV-2019 (92.8% a.a identity), but more distant from the SARS-CoV-1, bat-SL-CoV-Rp3 and bat-SL-CoV-RmYN02 (77.3, 72.2, and 73.5% a.a identity, respectively, Supplementary Table 1). In addition to the ORF9b, a predicted ORF9c retained in the SARS-CoV-1, bat SL-CoVs and Pangolin-CoV-2019 has also been described in the SARS-CoV-2 genome (Figure 4C). Dominguez Andres et al. (2020) report that the viral SARS-CoV-2 ORF9c protein (also named ORF14) is unstable and has acquired a transmembrane domain. They found that, when expressed in a human lung epithelial cell line (A549), the SARS-CoV-2 ORF9c interfered with IFN signaling, and several other pathways. A strong similarity is observed between the ORF9c proteins of SARS-CoV-2 (73 a.a), bat-SL-CoV-RatGT13 and Pangolin-CoV-2019, where the SARS-CoV-1 ORF9c protein appears to be closer to the ORF9c of bat-SL-CoV-Rp3.

### ORF10 Protein

Unlike the SARS-CoV-1, the bat-CoV-SL-RaTG13, the Pangolin-CoV-2019 and the SARS-CoV-2 possess a supplementary putative extra ORF named ORF10, which may encode for a protein of 38 a.a (Figure 4C and Supplementary Table 1). Recent studies demonstrated that very few transcripts containing ORF10 are detected in SARS-CoV-2-infected cells and several teams have reported that ORF10 is not a coding region (Caly et al., 2020; Davidson et al., 2020; Kim et al., 2020). The disease induced by the SARS-CoV-2 variant strains, in which the ORF10 gene was prematurely terminated, was not attenuated and *in vitro* replication analysis showed no difference compared to the wild-type virus (Jungreis et al., 2020; Pancer et al., 2020).

## Conclusion

Highly pathogenic coronaviruses have emerged regularly since the beginning of the 21st century, resulting in epidemics: SARS-CoV-1 in 2002–2003 and MERS-CoV in 2012 and a SARS-CoV-2 pandemic since 2019. The SARS-CoV-1, MERS-CoV and SARS-CoV-2 are all human coronaviruses closely related to bat-SL-CoVs. It can be tempting to speculate that the direct ancestor of these viruses may have arisen from sequential recombination events between the precursors of the bat-SL-CoVs prior to spillover to intermediate hosts or directly to humans. However, it is also important to note that after the SARS-CoV-1 outbreak in 2002–2003, bats were sampled extensively for coronaviruses and other viruses, although the latter probably represent a major initial reservoir. It is also possible that a similar, as yet unexplored, diversity of viruses exists in other animals. So, continuing surveillance in bats, but also in others animals (reservoir hosts or intermediate hosts), is vital to uncover the origin of these viruses but also to prevent the emergence of a new coronavirus disease. A better understanding of the bat coronavirus ecology, physiology and immunity response is also critical to elucidate the origin, the diversity and the emergence of coronaviruses. As we discussed above, the SARS-CoV-1, MERS-CoV, and SARS-CoV-2 encode several accessory proteins. Reverse genetic studies have demonstrated that none of them are essential for virus replication *per se*, but most of them appear to play a crucial role in virus pathogenesis. As the pathophysiological significances of these accessory proteins are poorly understood, further structural, evolution and functional studies are required to shed more-light on their precise functions and also to propose new antiviral targets. Increasing evidences show that preserving natural habitats reduces the risk of emergence or re-emergence of pathogens from wildlife. Ecosystems that are heavily used by humans (modern agriculture, deforestation, and urbanization) contain a tremendous variety of wildlife that carry pathogens (for many, still totally unknown) capable of infecting people. As the human population expands in an increasingly globalized world together with socio-economical changes, human contact with wildlife will continue inevitably to increase. This increases the risk of zoonotic viruses of emerge, including CoVs. To date, coronavirus surveillance has been almost entirely restricted to China. More vigorous field research efforts tracking the circulation of beta-CoVs more generally is needed across a broader global range if we are to avoid future repeats of the COVID-19 pandemic. Changes in our behaviors, but also integrative research across disciplines, by applying a “One Health” concept from field to lab, are urgently needed to prevent future zoonotic events and the repetition of the current episode.

## Author Contributions

Conceptualization of the article and writing of the original draft was done by NC.

## Conflict of Interest

The author declares that the research was conducted in the absence of any commercial or financial relationships that could be construed as a potential conflict of interest.

## Supplementary Material

Supplementary Table S1 Sequences were analyzed using Unipro UGENE: a unified bioinformatics tollkit Okonechnikov; Golosova; Fursov. Bioinformatics 2012 28: 1,166–1,167. For each ORF, the SARS-CoV-2 sequence (NC_045512) was used as a reference sequence to perform the alignment. Identity was calculated using SIM – Alignment Tool for protein sequences (www.expasy.org/).

Supplementary Table S2 Sequences were analyzed using Unipro UGENE: a unified bioinformatics tollkit Okonechnikov; Golosova; Fursov. Bioinformatics 2012 28: 1,166–1,167. For each ORF, the SARS-CoV-2 sequence (NC_045512) was used as a reference sequence to perform the alignment. Identity was calculated using SIM - Alignment Tool for protein sequences (www.expasy.org/).
