# Supplementary figures and images for: Coronavirus, the King Who Wanted More Than a Crown: From Common to the Highly Pathogenic SARS-CoV-2, Is the Key in the Accessory Genes?
Source: Front Microbiol. 2021 Jul 14;12:682603. doi: 10.3389/fmicb.2021.682603 (PMC8317507; doi:10.3389/fmicb.2021.682603)

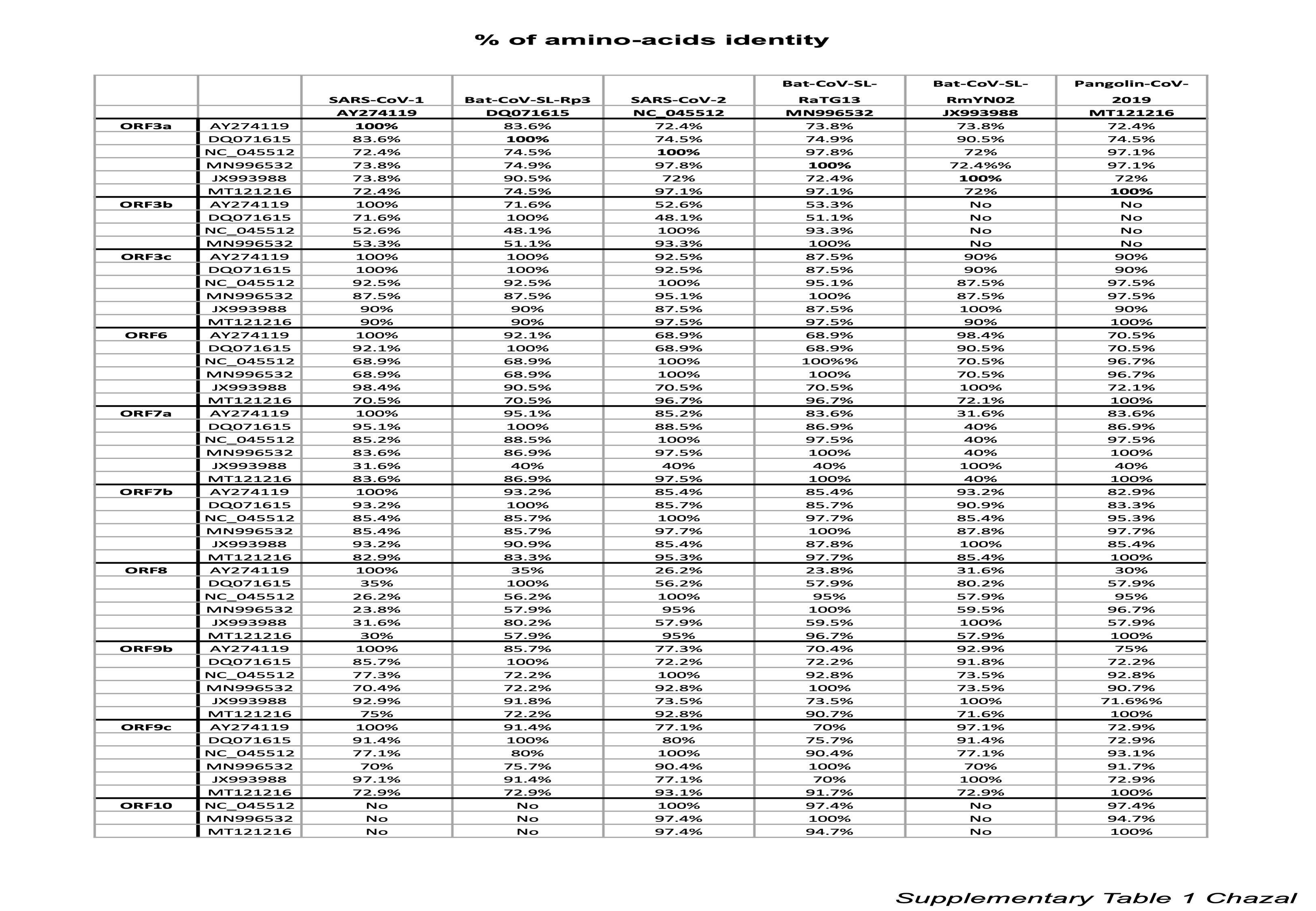

Supplement: Supplementary Table S1 — Sequences were analyzed using Unipro UGENE: a unified bioinformatics tollkit Okonechnikov; Golosova; Fursov. Bioinformatics 2012 28: 1,166–1,167. For each ORF, the SARS-CoV-2 sequence (NC_045512) was used as a reference sequence to perform the alignment. Identity was calculated using SIM – Alignment Tool for protein sequences (www.expasy.org/). [file Image_1.TIFF]

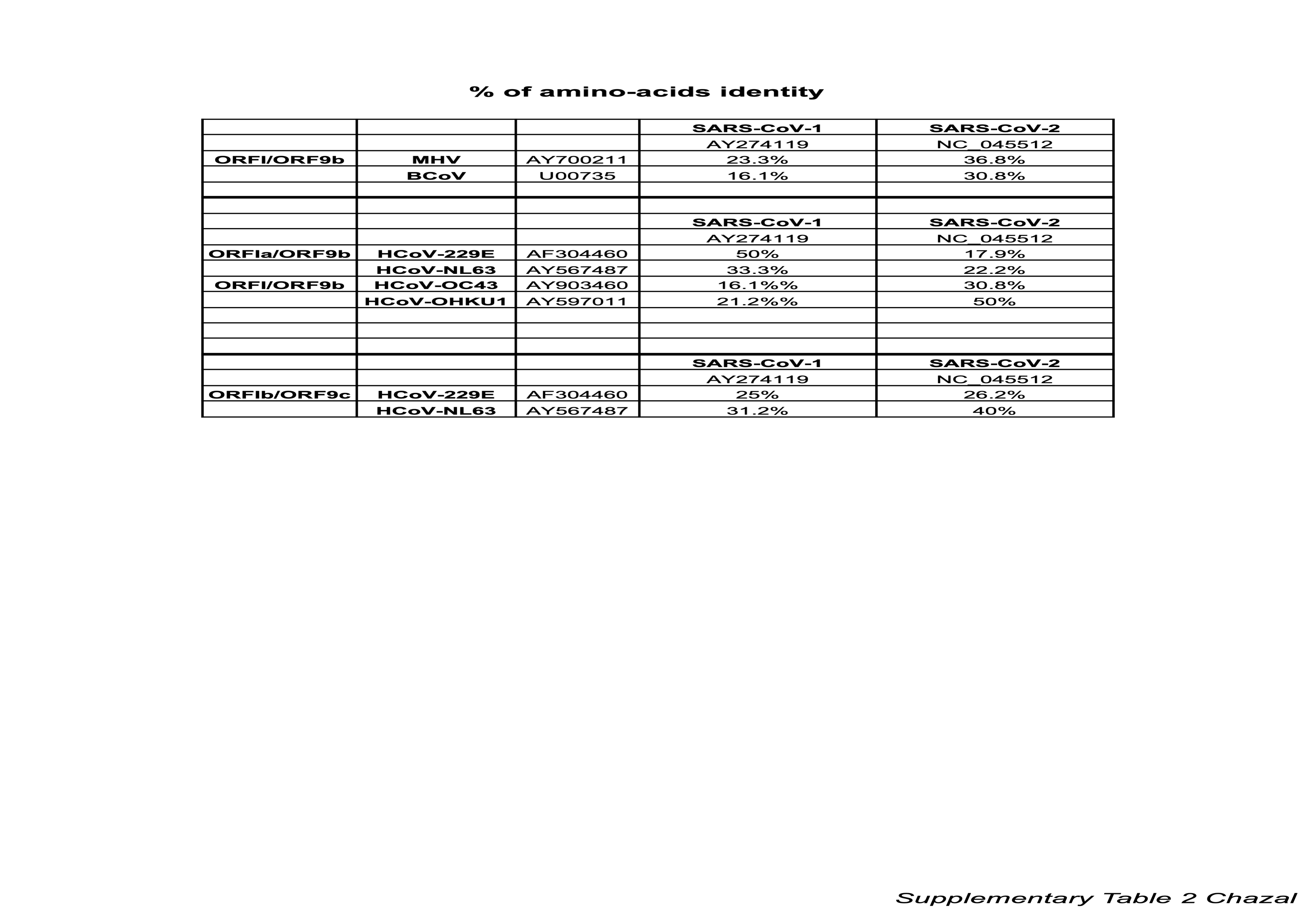

Supplement: Supplementary Table S2 — Sequences were analyzed using Unipro UGENE: a unified bioinformatics tollkit Okonechnikov; Golosova; Fursov. Bioinformatics 2012 28: 1,166–1,167. For each ORF, the SARS-CoV-2 sequence (NC_045512) was used as a reference sequence to perform the alignment. Identity was calculated using SIM - Alignment Tool for protein sequences (www.expasy.org/). [file Image_2.TIFF]
